# Facile Synthesis of Designer Shape-Defined Mesoporous Metal Nanoenzymes as Therapeutics for Diseases Involving Excessive Oxidative Stress

**DOI:** 10.34133/bmr.0251

**Published:** 2025-09-05

**Authors:** Xiongfeng Cao, Kun Chen, Minjun Ji, Xiang Liao, Yanfang Liu

**Affiliations:** ^1^ Department of Medical Imaging, Affiliated Hospital of Jiangsu University, Zhenjiang 212001, P. R. China.; ^2^ Institute of Imaging and Artificial Intelligence, Jiangsu University, Zhenjiang 212001, P. R. China.; ^3^ Center for Drug Research and Development, Guangdong Provincial Key Laboratory for Research and Evaluation of Pharmaceutical Preparations, Guangdong Pharmaceutical University, Guangzhou 510006, Guangdong, P. R. China.; ^4^ Department of Pharmacy, Affiliated Hospital of Jiangsu University, Zhenjiang 212001, P. R. China.; ^5^ Laboratory of Medical Imaging, The First People’s Hospital of Zhenjiang, Zhenjiang 212001, P. R. China.

## Abstract

Mesoporous metal nanomaterials (MMNs) have gained interest in biomedicine for their unique properties, but their potential is limited by the predominance of spherical shapes and the neglect of morphological effects on biological activity, which hinders the reasonable evaluation of morphology-dependent enzyme-like activities and biological behaviors and its further biomedical applications. It is therefore imperative to find an effective and facile method to design and prepare MMNs with novel, well-defined morphologies. Herein, we fabricated 3 mesoporous platinum nanoenzymes including sphere, rod, and bipyramid topologies [Au@mesoPt sphere, Au@mesoPt rod, and Au@mesoPt bipyramid nanoparticles (NPs), respectively] via a facile atomic layer deposition method using gold NPs (Au NPs) as the templated cores and Pluronic F127 as a structure-directing agent. The obtained Au@mesoPt NPs could enhance cellular uptake efficiency and prolong blood elimination half-lives, which helped more cancer cell spheroid permeation and accumulation at the disease sites post-injection. Au@mesoPt NPs could obviously alleviate atherosclerosis through reactive oxide species (ROS) scavenge due to its catalase-like activity and inhibition of pro-inflammatory cytokine release. Due to the role of metal nanoenzymes containing high-order-number (*Z*) elements as radiosensitizers, Au@mesoPt NPs have a distinct radiosensitizing on pancreatic cancer treatment. Among the shapes, Au@mesoPt bipyramids showed the best therapeutic efficacy in treating atherosclerosis and pancreatic cancer, likely due to their high aspect ratio, irregular surface, and anisotropy, which favor blood flow and cellular uptake. The tunable synthesis of shape-defined MMNs bodes well for other areas of application, including biosensors, surface-enhanced Raman scattering, surface plasmon resonance, hydrogen storage, catalysis, and electrotherapy.

## Introduction

With the uniquely large specific surface area and tunable pore volume/diameter, mesoporous nanomaterials (nanoscale materials with pore sizes on the order of 2 to 50 nm) currently were widely used in drug delivery [1], molecular imaging [2], biosensors [3], and tissue engineering [4]. Their unique chemical properties, which are largely a result of their large specific surface area and tunable pore volume/diameter, have stimulated interest for their potential translation to the clinic [5,6]. Notably, previous research has mainly focused on the properties of nonmetallic mesoporous nanomaterials (MMNs), with MMNs accounting for a relatively minor percentage of biomedical applications [7–9]. Compared with mesoporous pure nonmetallic nanomaterials [e.g., mesoporous silica nanoparticles (NPs)], MMNs possess the following advantages: (a) Superior optical and electrical properties. Compared with mesoporous pure nonmetallic nanomaterials, MMNs especially based on noble metal (Au, Ag, Pt, etc.) generally have excellent optical and electrical properties due to their strong surface-enhanced Raman scattering and electric conductivity, resulting in broader potential applications in optical sensing [10], electrochemical catalysis [11], and electronic devices [12]. (b) Higher enzyme-like activity. By virtue of the metal atom centers (including Mn [13], Fe [14], Au [15], Ag [16], Pt [17], etc.) present within their structure, MMNs commonly possess unique enzyme-like catalytic activities (such as redox, electrocatalysis, and photocatalysis enzyme-like activities) and selectivities not available to non-MMNs. For example, Pt-based mesoporous nanomaterials can severely react with hydrogen peroxide by their catalase-like activities [18]. (c) Imaging enhancement and radiosensitization effects. MMNs containing high-order-number (*Z*) elements (platinum or palladium, etc.) can serve as contrast agents for computed tomography and also as radiosensitizers to enhance the effects of cancer radiotherapy (RT) [9,18]. Due to their mesoporous structures, optical-electrical properties, enzyme-like activity, imaging enhancement, and radiosensitization effects, MMNs are receiving increased attentions in the field of nanomedicine. Specific applications have already been demonstrated in persistent tumor hypoxia relief [19], enhanced RT efficacy [20], potentiated immunotherapeutic therapies [21], as well as the treatment of excessive oxidative stress-associated diseases, such as cardiovascular atherosclerosis [22] or solid tumors [23], in which the high concentration of peroxides (e.g., hydrogen peroxide) and the remarkable decrease of reducing substances [e.g., glutathione (reduced form) (GSH)] are often observed.

Despite their impressive biological and physical activities, MMNs have largely been limited to only classical sphere or sphere-like morphologies. Previous studies and simulations have demonstrated that the biological properties of NPs, including cellular adhesion and uptake [24–26], blood circulation time [27], tissue permeability [28], and tissue accumulation [29], are intimately related to their physicochemical parameters (e.g., morphology, surface area, pore volume, and surface ligand coverage), with morphology-related parameters being particularly crucial, such as aspect ratio, anisotropic geometry, and radius of curvature. When compared to sphere-like shapes, the specific morphological characteristics of rod-like, bowl-like, or irregular shapes with high aspect ratio, anisotropic geometry, and large radius of curvature can improve the pharmacokinetic profile of NPs, increase specific organ accumulation, as well as accelerate phagocytosis, for example, by directly piercing into the cell membrane or increasing cell–material interactions [30–32]. However, current methodological limitations of obtaining MMNs with nonclassical shape prevent the exploration of these favorable biological properties in the context of MMNs. It is therefore imperative to develop effective, facile methods to prepare MMNs with biologically advantageous morphologies.

**Fig. 1.**
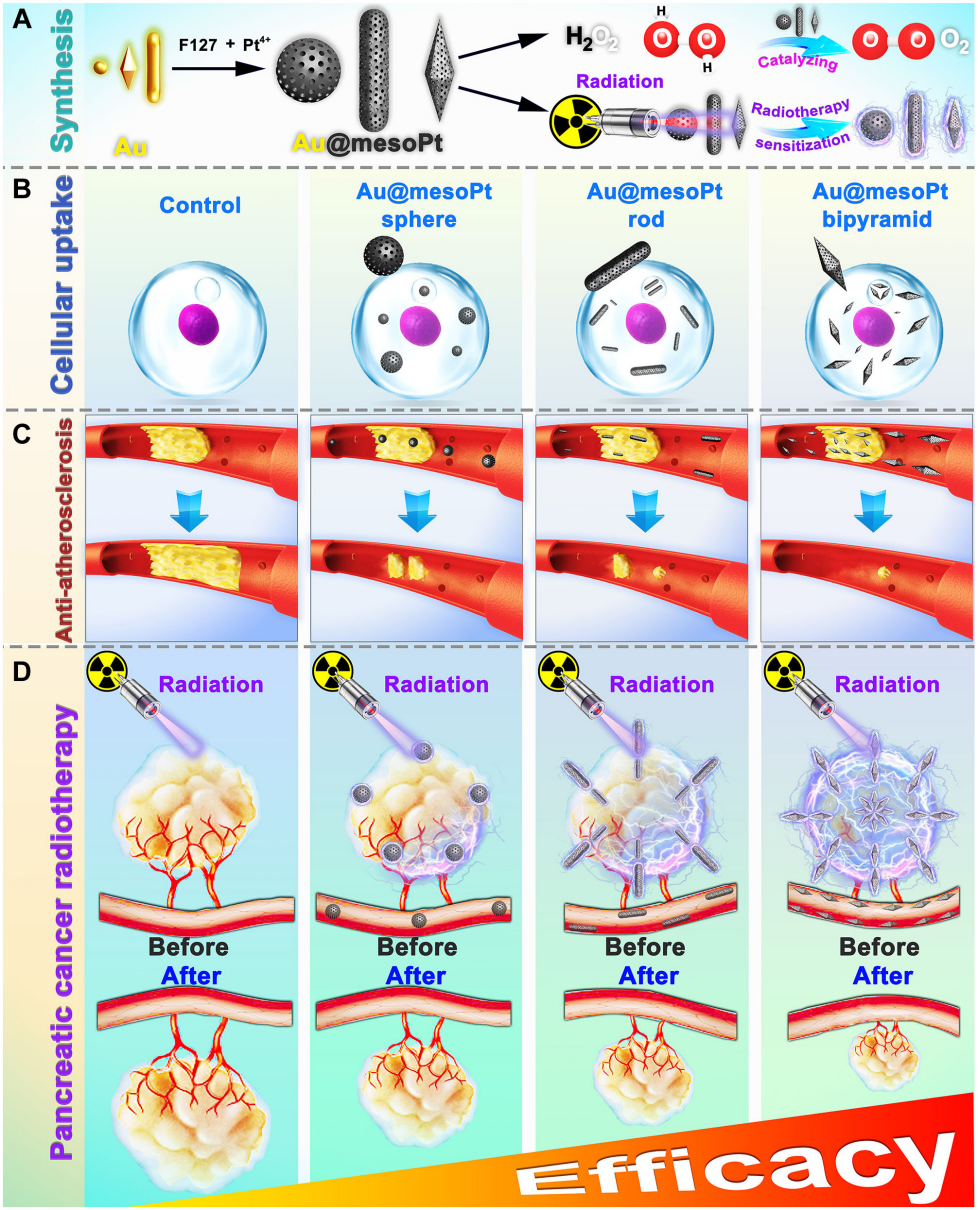
Schematic of Au@mesoPt NPs in synthesis (A), cellular uptake (B), anti-atherosclerosis therapy (C), and pancreatic cancer RT (D).

Herein, we report a suite of mesoporous platinum nanoenzymes with previously inaccessible morphologies, for use as therapeutics in disease models associated with excessive oxidative stress. This platform, consisting of sphere-, rod-, and bipyramid-shaped Au–Pt core–shell MMNs (designated Au@mesoPt sphere, Au@mesoPt rod, and Au@mesoPt bipyramid NPs, respectively), was constructed via atomic layer deposition (ALD) methods using Au NPs as the templated cores and Pluronic F127 as a structure-directing agent. The obtained metal nanoenzymes exhibited satisfactory catalase-like activity and showed distinct morphology-dependent differences in biological behaviors (Fig. 1A and B). Specifically, Au@mesoPt bipyramid possessed the highest cellular uptake efficiency at 6 h (39.31% cf. sphere: 13.06%, rod: 26.05%), the greatest cancer cell spheroid permeability (penetrated into the core of the cell spheroid for Au@mesoPt bipyramid), and the longest plasma elimination half-lives (*t*_1/2β_) (4,057.81 ± 142.83 min cf. sphere: 45.6 ± 1.8 min, rod: 73.22 ± 1.21 min). As a result, Au@mesoPt bipyramid showed the highest tumor accumulation at 24 h (12% cf. sphere: 6.9%, rod: 9.6%) and atherosclerotic effector cell (Raw 264.7) endocytosis at 6 h (98.88% cf. sphere: 93.26%, rod: 95.12%) and further achieved the best therapeutic efficacy in pancreatic cancer RT and the alleviation of atherosclerosis, both of which have been deemed as classical increased oxidative stress diseases (Fig. 1C and D). The potential reason for the improved biological properties of the Au@mesoPt bipyramid most likely stems from its increased aspect ratio, radius of curvature, and anisotropy compared with the sphere- and rod-shaped counterparts, a rationale that is supported by previous studies [30]. Overall, the successful preparation of diverse Au@mesoPt NPs in this work demonstrates a coherent strategy for the morphological regulation of MMNs, which enable further exploration of morphology-dependent effects in other metal nanoenzymes and metal-based hybrids.

## Materials and Methods

### Materials

Cetyltrimethylammonium bromide (CTAB), cetyltrimethylammonium chloride (CTAC), silver nitrate (AgNO_3_; >99%), gold (III) chloride trihydrate (HAuCl_4_·3H_2_O; ≥99.9%), chloroplatinic acid hexahydrate (H_2_PtCl_6_·6H_2_O; ≥99.95%), L-ascorbic acid (1-AA; ≥99%), sodium oleate (NaOL; >97%), Cyanine5.5 carboxylic acid (Cy5.5-COOH), N-hydroxysulfosuccinimide (NHS; 98%), 1-ethyl-3-(3-dimethyllaminopropyl) carbodiimide (EDC; 99%), concentrated hydrochloric acid (HCl; 9.7 wt %), Dulbecco’s modified Eagle’s medium (DMEM; 10 × 500 ml), singlet oxygen sensor green (SOSG), and ROS probe 2′,7′-dichlorodihydrofluorescein diacetate (DCFH-DA) were purchased from Aladdin Chemical (Shanghai, China). *N*,*N*-dimethylformamide (DMF; 37 wt %) was purchased from Sigma-Aldrich (Shanghai, China). Sodium borohydride (NaBH_4_; 96%), concentrated aqueous ammonia solution (NH_3·_H_2_O; 25 to 28 wt %), and anhydrous ethanol were obtained from Nanjing Chemical Reagent Co. Ltd. (Nanjing, China). Deionized water (18.2 MΩ cm) used in all experiments was from Millipore.

### Characterization methods

Transmission electron microscopy (TEM) images were obtained from the FEI TF20 microscope (Tokyo, Japan) at 100 kV. Scanning transmission electron microscope in high-angle annular dark field (STEM-HAADF) images used Hitachi S4800 microscope (Tokyo, Japan) at 5 kV. Fourier transform infrared (FT-IR) spectra were acquired from a NEXUS870 (Nicolet Instruments Inc., Madison, WI, USA) spectrometer. Ultraviolet–visible (UV–vis) absorption spectrum was measured utilizing a PerkinElmer's Lambda 35 UV–vis spectrophotometer (Perkin Elmer Inc., Massachusetts, USA). Hydrodynamic diameter and zeta potential were obtained by dynamic light scattering technology using a Zeta PALS analyzer (Brookhaven Instruments Co., Holtsville, USA). The concentration of gold ion and platinum ion was measured by inductively coupled plasma atomic emission spectroscopy (ICP-AES) spectrometer (PerkinElmer’s Optima5300DV). The photothermal images were captured by MAGNITY f15F1 infrared camera (Wuhan VST Light & Technology Co. Ltd., China). Nitrogen adsorption–desorption isotherms were obtained using an analyzer at −196 °C (Micromeritics ASAP 2020). Near-infrared (NIR) fluorescence images were captured by Interactive Video Information System (IVIS) Lumina XR imaging system under a Cy5.5 filter (λ_ex_ = 640 nm, λ_em_ = 705 nm). The laser source was an 808-nm diode pumped laser from Hi-Tech Optoelectronics Co. Ltd.

### Preparation of Au@mesoPt NPs

Au NPs, including Au sphere, Au rod, and Au bipyramid, were prepared according to the previous methods [33–35]. Geometric models and equations for calculating the molar concentration of Au NPs are provided in Fig. S1A to C. Briefly, the volumes of the Au NPs were calculated by measuring the associated widths and lengths via TEM, followed by quantification according to the results of ICP-AES analysis.

ALD method: Pluronic F127 (90 mg), KBr (200 mg), and ascorbic acid (53 mg) were added sequentially to a solution of the appropriate Au NPs (5.5 ml, sphere: 0.35 nM, rod: 1.75 nM, bipyramid: 10.5 nM) and allowed to dissolve. Afterward, H_2_PtCl_6_ (150 μl of 0.2 M stock solution) was added, and the solution was thoroughly mixed (shaken by hand) before being aged at 70 °C for 12 h. The resultant precipitate was isolated by centrifugation (10,000 rpm, 10 min) and washed twice with distilled water. For the further surface modifications and enhanced biocompatibility, the Au/Pt NPs were PEGylated by dispersing the material in 5 ml of NH_2_-PEG-SH (molecular weight = 1,000 Da, 3 mg ml^−1^), followed by shaking (250 rpm) at room temperature for 12 h. Finally, the mixture was washed 7 times with water (centrifuge, 10,000 rpm, 10 min) to remove residual NH_2_-PEG-SH and F127. The final products (Au@mesoPt sphere, Au@mesoPt rod, and Au@mesoPt bipyramid) were dispersed in 10 ml of distilled water for further use.

### Preparation of Cy5.5-modified Au@mesoPt NPs

Cy5.5-COOH (400 μl of 5 mg l^−1^ DMF stock solution) was added to a solution of NHS (5 ml of 20 mg ml^−1^ DMF stock solution) and EDC (5 ml of 20 mg ml^−1^ DMF stock solution), followed by shaking at room temperature for 6 h. Subsequently, the appropriate Au@mesoPt NP (10 ml, 7.59 pM) was added, followed by shaking (250 rpm) at room temperature for 12 h. The product was then washed by centrifugation 3 times with water (10,000 rpm, 10 min) before the resulting Cy5.5-modified NP precipitate was redispersed in phosphate-buffered solution (PBS; 2 ml) for further use.

### Cell culture and cytotoxicity

Human pancreatic cancer cell line (Aspc-1) and murine-derived macrophage (Raw 264.7), purchased from Shanghai Bioray Biotechnology Co. Ltd., were cultured in DMEM with 10% fetal bovine serum (FBS) and 1% penicillin–streptomycin in an incubator (humidified atmosphere, 5% CO_2_, 37 °C). To evaluate the cytotoxicity of the Au@mesoPt NPs, Aspc-1 cells or Raw 264.7 cells were seeded in 96-well plates at a density of 1 × 10^4^ cells per well. After overnight incubation, DMEM solution of the desired NPs dispersed (0 to 10 pM) was added to the cells and allowed to incubate (48 h, 37 °C). Following this, the culture medium in each well was replaced with fresh DMEM (110 μl) containing Cell Counting Kit-8 (CCK-8 ) formazan solution (10 μl) and then incubated for 1 h. The absorbance was measured using a microplate reader (Tecan, Switzerland) at 450 nm. Cell viability was calculated using the following formula: Cell viability (%) = [(*A*_e_ − *A*_c_)/(*A*_0_ − *A*_c_)] × 100, where *A*_e_ is the treatment group absorbance, *A*_c_ is the control group absorbance (no cells), and *A*_0_ is the control group absorbance (untreated cells).

### In vitro intracellular distribution of Au@mesoPt NPs

Aspc-1 cells (5 × 10^4^ cells per well) were seeded on a 10-mm round coverslip placed in 12-well plates. After overnight incubation, the cells were co-incubated with Cy5.5-functionalized Au@mesoPt NPs (10 pM) for 1, 3, and 6 h at 37 °C. The cells were then washed with PBS (2 × 2 ml) and fixed with 4% formalin solution for 15 min. Finally, the cells were again washed with PBS (2 × 2 ml) and stained with 4′,6-diamidino-2-phenylindole (DAPI), followed by examination using confocal laser scanning microscope (CLSM; Leica, Germany).

### Cellular uptake of Au@mesoPt NPs in Aspc-1 and Raw 264.7 cells

The appropriate cells (Aspc-1 or Raw 264.7) were initially seeded in 12-well plates at a density of 1 × 10^5^ cells per well and incubated overnight. Following this, the cells were cocultured with freshly prepared Cy5.5-functionalized Au@mesoPt NPs (2 ml of 10 pM). After incubation for 0, 1, 3, and 6 h, the cells were washed with PBS (2 × 2 ml) and collected for analysis by flow cytometry [fluorescence-activated cell sorting (FACS); Beckman Coulter, NJ, USA].

### In vitro 3D multicellular spheroid penetration capability assay

Agarose (150 mg) was dissolved in hot (~90 °C) FBS-absent DMEM (10 ml) and added to a 96-well plate (100 μl per well). After naturally cooling to room temperature, Aspc-1 cells (1 × 10^4^ per well) were seeded into the agarose-bottomed 96-well plates, followed by culture in an incubator (37 °C, humidified atmosphere, 5% CO_2_). Once the diameter of the multicellular spheroids (MCSs) reached 600 to 700 μm, Cy5.5-functionalized Au@mesoPt NPs were introduced (100 μl per well, 10 pM DMEM stock solution). After incubation for 3 h, MCSs were washed twice with PBS and imaged using CLSM.

### In vivo toxicity

All animal experiments complied with the Animal Research: Reporting of In Vivo Experiments (ARRIVE 2.0) guidelines and were carried out in accordance with the National Institutes of Health Guide for the Care and Use of Laboratory Animals [36]. All procedures involving animals were performed under the guidelines approved by the Committee on the Use of Live Animals for Teaching and Research of Jiangsu University (no. UJS-IACUC-AP-2024031411).

To evaluate the in vivo toxicity of Au@mesoPt sphere, Au@mesoPt rod, or Au@mesoPt bipyramid, 20 nude mice were randomly divided into 4 groups (*n* = 5) and treated with PBS (100 μl per mouse) or Au@mesoPt NPs (100 μl per mouse, 50 nM kg^−1^), respectively. After 14 d, the blood samples and major organs (i.e., heart, liver, spleen, lung, kidney, and tumor) were collected for further analysis.

### Pharmacokinetic profiles

Fifteen nude mice were randomly divided into 3 groups (*n* = 5) and intravenously injected with the relevant Au@mesoPt NP (sphere, rod, or bipyramid, 100 μl per mouse, 50 nM kg^−1^). Blood samples were collected from caudal vein at the indicated time points (0, 1 min, 3 min, 5 min, 10 min, 15 min, 30 min, 1 h, and 3 h) post-injection, followed by digestion of blood samples using fresh aqua regia and measurement of Au and Pt concentration using ICP-AES.

### In vivo biodistribution

ICP-AES analysis and NIR fluorescence imaging were used to investigate the biodistribution of Au@mesoPt NPs in vivo. Firstly, 1 × 10^7^ Aspc-1 cells (dispersed in 100 μl of PBS) were injected into the subcutaneous tissue of 80 nude mice. After the tumor volume reached 150 mm^3^, tumor-bearing mice were randomly divided into 16 groups (*n* = 5 for each group). For ICP-AES analysis, nude mice were intravenously injected with PBS (100 μl per mouse), Au@mesoPt sphere, Au@mesoPt rod, or Au@mesoPt bipyramid (100 μl per mouse, 50 nM kg^−1^), respectively. For ex vivo NIR fluorescence imaging, nude mice were intravenously injected with PBS (100 μl per mouse) or Cy5.5-modified NPs (100 μl per mouse, 50 nM kg^−1^). After 12 or 24 h, the mice were sacrificed, major organs (heart, liver, spleen, lung, kidney, and tumor) were harvested, and the ex vivo NIR fluorescence imaging of Au mesoPt-Cy5.5 NPs in major organs was carried out with an IVIS. The organs were further digested using fresh aqua regia, and the Au and Pt concentrations were measured by ICP-AES analysis.

### Simulation method

The numerical simulation for the flow performance of the 3 Au@mesoPt NPs (Au@mesoPt sphere, Au@mesoPt rod, or Au@mesoPt bipyramid) in micro-blood vessels was performed by COMSOL Multiphysics simulation software (version 6.1, COMSL Inc., Sweden), containing most physical field partial differential equations for powerful multi-physics coupling capabilities. Two-dimensional (2D) planar fluid–structure coupling numerical simulation was established for the 3 NPs. The 3D geometric models of the 3 NPs were shown in Fig. S1D.

Considering the NPs flowing in laminar through a single blood vessel or microvessel, 2D models with the laminar flow application module can provide sufficient simulation accuracy. In fluid dynamics, Mach number was a crucial fluid parameter, equaling to the ratio of flow velocity to the speed of sound. The smaller Mach number (generally less than 0.3 for most biological fluids; e.g., blood flow) disclosed, the slower the fluid velocity. Due to its low-density variation, the compressibility effect of blood flow could be neglected, ensuring feasible analysis for the flow change of Au@mesoPt NPs in established blood flow model by postprocessing. The flow equations were as follows:

$$\rho \frac{\partial U}{\partial t}+\rho \left(u\cdot \nabla \right)u=\nabla \cdot \left[-\mathrm{pI}+k\right]+F+\rho g$$ (1)

ρ∇·u=0 (2)

$$K=\mu \left(\nabla \cdot u+{\left(\nabla \cdot u\right)}^{T}\right)$$ (3)

The above equations were fundamental equations in fluid mechanics, commonly known as the Navier–Stokes equations. *ρ* represents the density of the fluid. *u* represents the velocity field. *g* represents gravitational acceleration. *t* represents time. ∇ represents the spatial gradient operator. *p* represents the fluid pressure. *I* represents unit-matrix. *k* represents the stress tensor, describing the viscous stress of the fluid and determined by fluid viscosity *μ* and the gradient of *u*. Eq. 1 was the momentum conservation equation, describing how the momentum of the fluid changes with time and space. Eq. 2 was the continuity equation, also known as the mass conservation equation, describing how the density of the fluid changes with time and space. Eq. 3 was the definition of the stress tensor, defining the relationship among the stress tensor *k*, the fluid viscosity *μ*, and the gradient of the velocity field *u*.

Solid mechanics control equations: The stress field in this model was calculated by using the plane strain approach, specifically employing the solid mechanics application module to handle the solid particle portion. The Au@mesoPt NPs were deemed as linear elastic solid materials, and the solid mechanics equations were as follows:

$$\rho \frac{\partial^{2}u_{2}}{\partial t^{2}}=\nabla \cdot {\left(F\cdot S\right)}^{T}+F_{v}$$ (4)

$$F=I+\nabla u_{2}$$ (5)

The above equations were partial differential equations describing the motion of elastic solids and also known as fundamental motion equations in solid mechanics. In the equations, *ρ* represented the mass density of the solid, $u_{2}$ represented the displacement vector of the solid, *t* represented time, ∇ represented the gradient operator representing spatial variations and *S* was the Piola−Kirchhoff stress tensor, describing the internal stress state of the object. F_v_ was the volume force (such as gravity). *I* represented unit-matrix. *F* was the deformation gradient tensor representing the deformation of the object. *F* = *I* meant that the object was not deformed. *F* ≠ *I* meant that the object has undergone deformation.

The left part of Eq. 4 was the rate of momentum change of the solid (i.e., the mass times the acceleration), and the right part was the sum of all the forces acting on the solid. The physical meaning of this equation was Newton’s second law: The force acting on an object was equal to the rate of momentum of the object. The rate of momentum change of solids generally was described by the product of the second time derivative (acceleration) of the displacement and the mass density. The applied force included the stress-induced force and the volume force.

The boundary and the initial conditions: In the model of simulating Au@mesoPt NPs moving along blood vessel, the left side of pipeline was set as the entrance of blood vessel, and the right side was export. The residual parts were designated as the blood vessel walls (Fig. S1E).

Mesh partitioning: In order to make the numerical simulation results more accurate, free quadrilateral mesh and computational fluid dynamics (CFD) mesh calibration were used. Considering that the dynamic mesh changes continuously during the flow process may lead to a decrease in mesh quality, automatic re-meshing was added in the solver to optimize this problem. A new mesh with higher quality would be automatically redrawn when the mesh quality was lower than the set threshold. The initial mesh after partitioning was shown in Fig. S1F.

### Evaluating catalase-like activity of Au@mesoPt NPs

PBS (5 ml), Au@mesoPt sphere (5 ml, 10 pM), Au@mesoPt rod (5 ml, 10 pM), and Au@mesoPt bipyramid (5 ml, 10 pM) were added to 4 separate 15-ml centrifuge tubes with H_2_O_2_ (final concentration of 3.0 wt %) at room temperature and then monitored for O_2_ bubble generation. The centrifuge tubes were periodically photographed in order to quantify this. PBS (100 μl), Au@mesoPt sphere (100 μl, 10 pM), Au@mesoPt rod (100 μl, 10 pM), and Au@mesoPt bipyramid (100 μl, 10 pM) were added in a 96-well plate with H_2_O_2_ (final concentration of 3.0 wt %) and [(Ru(dpp)_3_)]Cl_2_ (10 μg ml^−1^, 10 μl) and incubated at 37 °C for 5 min. The emission at 620 nm was recorded for each well after excitation at 488 nm.

### Relieving lipopolysaccharide-induced ROS in Raw 264.7 cells

Raw 264.7 cells (1 × 10^5^ cells per dwell) were seeded into a 96-well plate and incubated overnight. Then, the cells were stimulated with or without 200 ng ml^−1^ of lipopolysaccharide (LPS) (100 μl per well) and further allowed to incubate for 12 h, before being washed once with PBS (100 μl per well). The treated cells were incubated with DMEM (100 μl per well) containing DCFH-DA (20 μM) for a further 4 h. Afterward, the cells were washed once with PBS and incubated with either DMEM (100 μl), Au@mesoPt sphere (100 μl, 10 pM), Au@mesoPt rod (100 μl, 10 pM), or Au@mesoPt bipyramid (100 μl, 10 pM) for 6 h. Subsequently, the cells were washed once with PBS and the fluorescence emission was recorded at 530 nm (excited at 485 nm) using a microplate reader (Infinite M200 pro, Tecan, Switzerland).

### Pro- and anti-inflammatory cytokine detection

Raw 264.7 cells (1 × 10^6^ cells per well) were seeded into a 6-well plate. After incubation overnight, the cells were stimulated with 200 ng ml^−1^ of LPS (2 ml per well) or left untreated and incubated for 12 h. Then, the cells were washed once with PBS and then co-incubated with DMEM (2 ml per well), Au@mesoPt sphere (2 ml per well, 10 pM), Au@mesoPt rod (2 ml per well, 10 pM), or Au@mesoPt bipyramid (2 ml per well, 10 pM) for a further 4 h. Finally, the cell supernatants were collected, with interleukin-6 (IL-6), tumor necrosis factor-α (TNF-α), IL-10, and transforming growth factor-β (TGF-β) levels quantified using enzyme-linked immunosorbent assay (ELISA) analysis (all from Elabscience Biotech) according to the vendor’s instructions.

### Ex vivo NIR fluorescence imaging of aorta in atherosclerosis animal model

The atherosclerosis mice were developed by high cholesterol western type diet feeding of APOE^−/−^ mice for 12 weeks according to the previous research [37]. Twelve atherosclerotic mice were randomly divided into 4 groups (*n* = 3), including (a) PBS group, (b) Au@mesoPt sphere-Cy5.5 NP group, (c) Au@mesoPt rod-Cy5.5 NP group, and (d) Au@mesoPt bipyramid-Cy5.5 NP group, and administered the appropriate treatment (100 μl of PBS, 50 nM kg^−1^ NPs). After 4-h injection, all mice were euthanized, and the aorta was collected for ex vivo NIR fluorescence imaging and then fixed in 4% formalin solution for histological analysis.

### Therapeutic effect in atherosclerosis animal model

Atherosclerotic mice (*n* = 20) were randomly divided into 4 groups (*n* = 5 per group), including (a) PBS group, (b) Au@mesoPt sphere group, (c) Au@mesoPt rod group, and (d) Au@mesoPt bipyramid group, and intravenously injected with the appropriate treatment (100 μl of PBS, 50 nM kg^−1^ NPs) once a day for 2 weeks. Then, the mice were continuously monitored for 2 weeks. During the treatment and monitoring periods, all mice remained on a high cholesterol western type diet. All mice were then euthanized, and the aorta was collected and fixed in 4% formalin solution for histological analysis.

### In vitro ROS generation of Au@mesoPt NPs under x-ray irradiation

Singlet oxygen (^1^O_2_) was detected with the widely used probe of singlet oxygen sensor green (SOSG). In the presence of ^1^O_2_, the intrinsic fluorescence of SOSG was restored, which resulted in increased fluorescence. PBS (100 μl), Au@mesoPt sphere, Au@mesoPt rod, Au@mesoPt bipyramid solution (100 μl, 10 pM), and SOSG (50 × 10^−6^ M, 10 μl) were mixed in 4 separate 1.5-ml centrifuge tubes with or without H_2_O_2_ (final concentration of 3.0 wt %) at room temperature, and they were treated with or without x-ray irradiation (8 Gy). The fluorescence intensity was detected using a microplate reader (Infinite M200 Pro, Tecan, Switzerland) (λ_ex_ = 490 nm, λ_em_ = 530 nm) 30 min after x-ray irradiation.

2′,7′-Dichlorodihydrofluorescein diacetate (DCFH-DA) was selected as a probe to detect the generation of intracellular ROS. Aspc-1 cells (1 × 10^4^ cells per well) were initially seeded in 96-well plates, with 5 wells were performed in parallel for each concentration. After overnight incubation, either DMEM (100 μl containing 20 μM DCFH-DA) or an Au@mesoPt NP solution (100 μl per well, 10 pM in DMEM containing 20 μM DCFH-DA) was added and allowed to co-incubate for 6 h at 37 °C. After washing with PBS (2 × 100 μl), fresh DMEM (100 μl per well) was introduced and then treated with or without x-ray irradiation (8 Gy). Subsequently, the cells were washed once with PBS and the fluorescence emission was recorded at 530 nm (excited at 485 nm) using a microplate reader (Infinite M200 Pro, Tecan, Switzerland).

### In vitro enhancement of RT by Au@mesoPt NPs

Aspc-1 cells (1 × 10^4^ cells per well) were initially seeded in 96-well plates, with 5 wells performed in parallel for each concentration. After overnight incubation, either DMEM or an Au@mesoPt NP solution (100 μl per well, 10 pM in DMEM) was added and allowed to co-incubate for 12 h at 37 °C. After washing with PBS (2 × 100 μl), fresh DMEM (100 μl per well) was introduced and then treated with or without x-ray irradiation (8 Gy). CCK-8 formazan solution (10 μl) was then added, and the culture was further incubated for 1 h. Finally, the absorbance value was measured using a microplate reader (Tecan, Switzerland) at 450 nm, and cell viability was calculated using the previous formula (see the Cell culture and cytotoxicity section).

Cell live/dead staining assay: Aspc-1 cells (5 × 10^4^ cells per well) were seeded in 12-well plates. After overnight incubation, the cells were co-incubated with Au@mesoPt NPs (2 ml of 10 pM) for 12 h. After washing with PBS (2 × 2 ml), fresh DMEM (1 ml per well) was added and the mixture was treated with or without x-ray irradiation (8 Gy). Cell live/dead staining was performed following the manufacturer’s instructions (Invitrogen), with live cells stained green and dead cells stained red. The fluorescence images were captured by an IX71 microscopy (Olympus).

### In vivo RT

Aspc-1 xenograft pancreatic cancer models (40 nude mice) were established as described above. Once the tumor volume reached 100 mm^3^, tumor-bearing mice were randomly divided into 8 groups (*n* = 4 for each group). These include (1) PBS group, (2) Au@mesoPt sphere group, (3) Au@mesoPt rod group, (4) Au@mesoPt bipyramid group, (5) RT group, (6) Au@mesoPt sphere + RT, (7) Au@mesoPt rod + RT, and (8) Au@mesoPt bipyramid + RT group, followed by intravenously injecting with PBS (100 μl per mouse) in groups 1 and 5 or NPs (100 μl per mouse, 50 nM kg^−1^) in groups 2 to 4 and 6 to 8. One day after administration (24 h), the tumor sites of the appropriate groups (5, 6, 7, and 8) were treated with x-ray irradiation (8 Gy) every other day for 3 times. Tumor sizes and mice body weight were then recorded every 2 d (from day 0 to day 14) for all mice, with tumor size being recorded as the maximum width (*a*) and the maximum length (*b*). The tumor volume was calculated using the following equation: tumor volume = *a*^2^*b*/2. After 14 d, all tumors were collected for histological analysis.

### Statistical analysis

All experimental results were analyzed by using GraphPad Prism 6 (version 6.01, GraphPad Software Inc., USA). Pharmacokinetic results were analyzed by Drug and Statistics (DAS) version 2.0. Fluorescence intensity and gray value were measured using ImageJ. Data analysis was performed by using Student’s *t* tests or one-way analysis of variance (ANOVA), followed by expression as mean ± the standard error of the mean (SEM). Statistical significance (*P < 0.05, **P < 0.01, ***P < 0.001) and no significance (NS) are as labeled in figures.

## Results and Discussion

### Preparation and characterization of Au@mesoPt NPs

It has been reported that the shape of a drug carrier can modulate its circulation in the blood, in vivo distribution, cellular uptake, and clearance via mononuclear phagocyte system [38]. Therefore, in this study, mesoporous Au–Pt core–shell NPs (termed as Au@mesoPt NPs) with different morphologies that regulate oxidation levels were fabricated, particularly focusing on rod-like and bipyramid-like topologies that were previously unexplored, to investigate therapeutic effects of various topological NPs on diseases involving excessive oxidative stress.

**Fig. 2.**
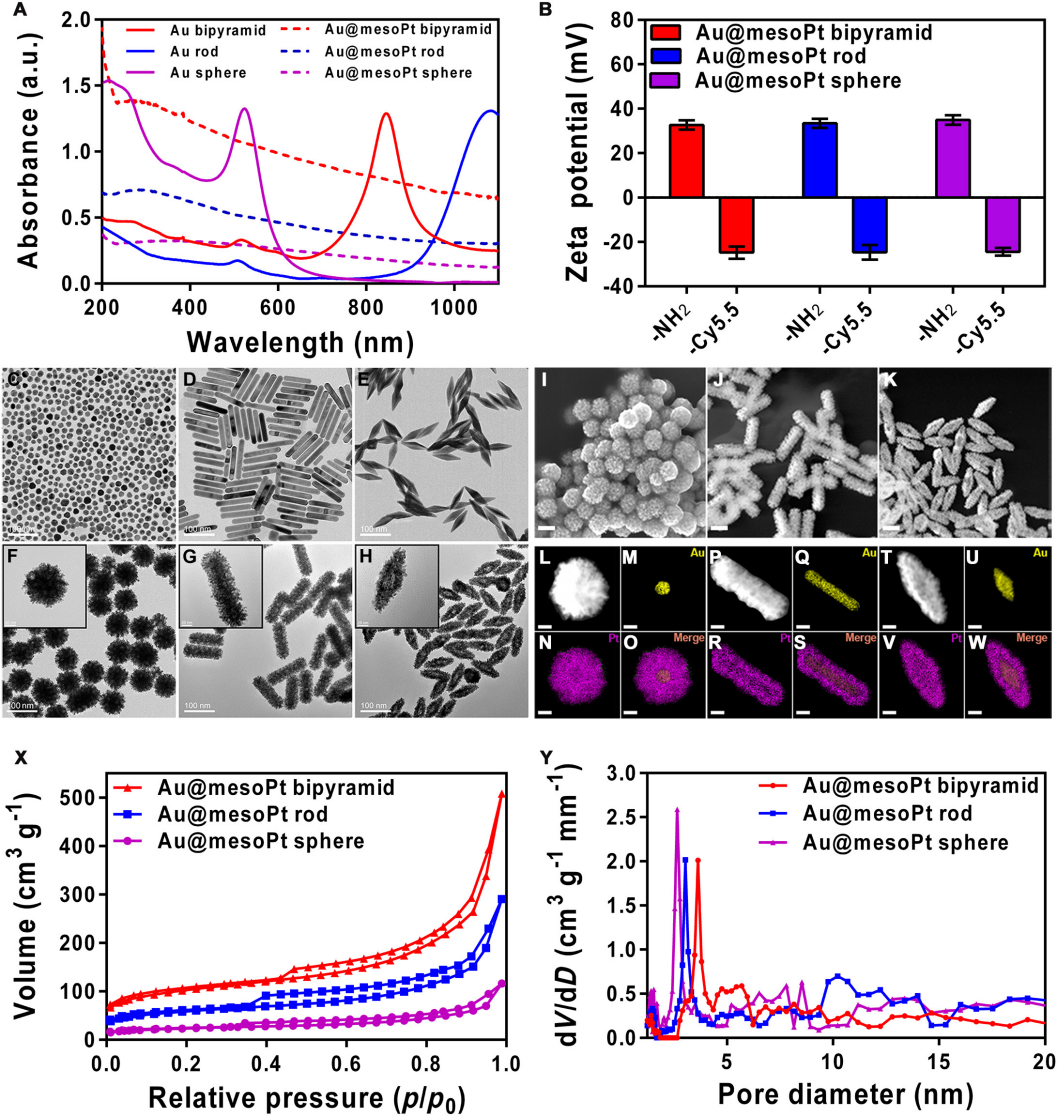
Characterization of the Au@mesoPt NPs. (A) UV–vis–NIR absorbance spectra of Au sphere, Au rod, Au bipyramid, Au@mesoPt sphere, Au@mesoPt rod, and Au@mesoPt bipyramid at an identical concentration of 5 pM. (B) Zeta potential of Au@mesoPt sphere, Au@mesoPt rod, and Au@mesoPt bipyramid modified with SH-PEG-NH_2_ and Cy5.5-COOH. (C to H) TEM images of Au sphere (C), Au rod (D), Au bipyramid (E), Au@mesoPt sphere (F), Au@mesoPt rod (G), and Au@mesoPt bipyramid (H). (I to K) SEM images of Au@mesoPt sphere (I), Au@mesoPt rod (J), and Au@mesoPt bipyramid (K). Sale bar, 100 nm. (L to W) STEM-HAADF images and EDX elemental mapping images of Au@mesoPt sphere (L to O), Au@mesoPt rod (P to S), and Au@mesoPt bipyramid (T to W). Scale bar, 20 nm. (X) Nitrogen adsorption–desorption isotherms of Au@mesoPt sphere, Au@mesoPt rod, and Au@mesoPt bipyramid. (Y) Pore-size distribution curves of Au@mesoPt sphere, Au@mesoPt rod, and Au@mesoPt bipyramid.

First, Au@mesoPt NPs were obtained using presynthesized, well-defined Au NPs as shape-directing templates, with the mesoporous platinum shell being subsequently added by ALD. The calculated Au NP sizes were in a good accordance with the observed model (Fig. S1A to D), indicating the Au NP templates possessed homogeneous morphologies suitable for further elaboration. The successful coating Pt shell on Au NPs core was first proven via UV–vis spectra. The absorption spectra of the naked Au particles and Au@mesoPt NPs showed that the typical absorption peaks of Au NPs (spheroidal: 520 nm, rod-like: 1,080 nm, double-pyramid: 845 nm; Fig. 2A) disappeared after coating with mesoporous Pt shell, which was attributed to the Pt layer blocking incident light absorbance of Au core. The absorbance of Au@mesoPt bipyramid (5 pM) was significantly stronger than identical concentration of Au@mesoPt sphere and Au@mesoPt rod, suggesting better light absorption ability of Au@mesoPt bipyramid than Au@mesoPt sphere and Au@mesoPt rod (Fig. 2A). Moreover, the standard absorbance curves of Au@mesoPt sphere, Au@mesoPt rod, and Au@mesoPt bipyramid exhibited apparently linear correlation between absorbance and concentration (*R*^2^ = 1, *R*^2^ = 0.995, and *R*^2^ = 0.9987, respectively), which enabled the accurate calculation of Au@mesoPt NP solution concentration (Fig. S2A to F). Additionally, zeta potential of Au@mesoPt sphere, Au@mesoPt rod, and Au@mesoPt bipyramid modified with SH-PEG-NH_2_ and Cy5.5-COOH was measured (Fig. 2B). All 3 Au@mesoPt NPs modified with SH-PEG-NH_2_ showed a net positive charge, with Au@mesoPt bipyramid NPs being the highest (sphere: 32.7 ± 1.9 mV, rod: 33.4 ± 1.8 mV, and bipyramid: 34.9 ± 1.9 mV), which suggested that these NPs would undergo efficient cellular uptake and tissue penetration. The 3 Au@mesoPt NPs modified with Cy5.5-COOH showed a net negative charge (sphere: −24.4 ± 0.8 mV, rod: −24.7 ± 1.5 mV, and bipyramid: −24.8 ± 1.2 mV). Furthermore, TEM images verified that the 3 distinct gold templates (Au sphere, Au rod, and Au bipyramid NPs) had been synthesized with smooth surfaces and uniform morphology (Fig. 2C to E). Following this, coating the gold templates with the mesoporous Pt shell efficiently generated the target materials, which possessed visibly rough surfaces, generally unordered mesoporosity, and uniform dispersity (sphere: ~85 × 85 nm, rod: ~116 × 38 nm, and bipyramid: ~99 × 38 nm; Fig. 2F to H). SEM images displayed obvious mesoporous structures on the surface of these 3 NPs (Fig. 2I to K). Energy-dispersive x-ray (EDX) elemental mapping images and EDX line profiles further confirmed the core–shell hybrid structure composition with Au in the core and Pt located in the outside range of Au@mesoPt sphere, Au@mesoPt rod, and Au@mesoPt bipyramid NPs (Fig. 2L to W and Fig. S3A to C). Subsequently, nitrogen adsorption–desorption isotherms were used to study the pore-size distribution of Au@mesoPt NPs. The results showed that Au@mesoPt sphere, Au@mesoPt rod, and Au@mesoPt bipyramid exhibited type IV curves with sharp capillary condensation steps in the *p*/*p*_0_ range of 0.3 to 0.4, 0.3 to 0.4, and 0.4 to 0.5, respectively, suggesting typical characteristic of the mesoporous NPs with narrow pore-size distribution (Fig. 2X). The surface area, pore volume, and pore size of Au@mesoPt bipyramid were calculated to be 291 m^2^ g^−1^, 0.53 cm^3^ g^−1^, and 3.6 nm, respectively, as well as Au@mesoPt sphere to be 207 m^2^ g^−1^, 0.33 cm^3^ g^−1^, and 2.6 nm and Au@mesoPt rod to be 255 m^2^ g^−1^, 0.43 cm^3^ g^−1^, and 3.0 nm, respectively, indicating obvious mesoporous structures of the NPs (Fig. 2Y). Therefore, these results confirmed the successful preparation of 3 different mesoporous structures of Au@mesoPt NPs, which might undergo efficient cellular uptake and tissue penetration.

### In vitro biological behaviors of Au@mesoPt NPs

**Fig. 3.**
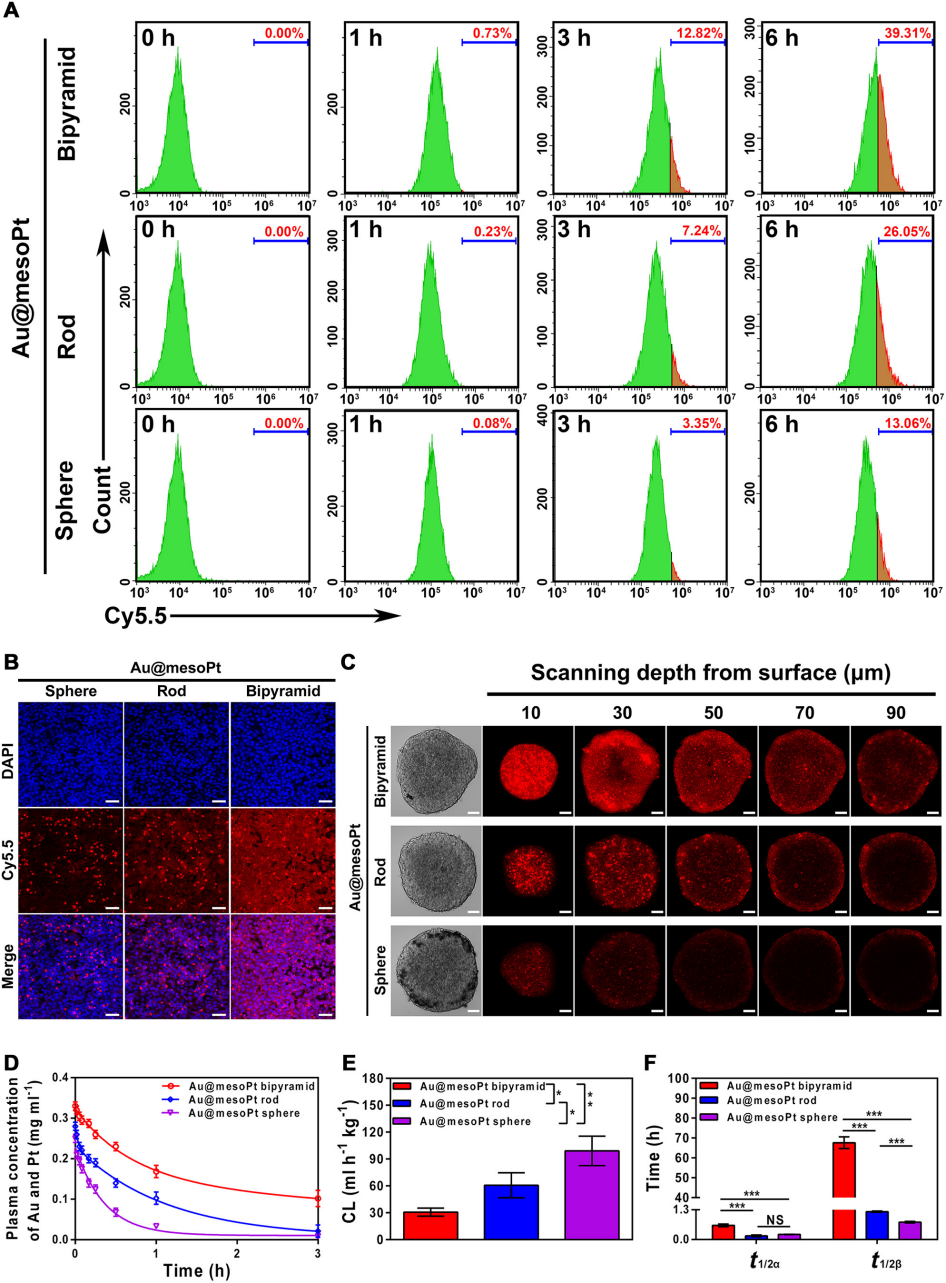
In vitro and in vivo biological behaviors of the Au@mesoPt NPs. (A) Flow cytometry analysis of Aspc-1 cell uptake after incubation with Au@mesoPt sphere, Au@mesoPt rod, and Au@mesoPt bipyramid at indicated times (1, 3, and 6 h). (B) CLSM images of Aspc-1 cells incubated with Au@mesoPt sphere, Au@mesoPt rod, and Au@mesoPt bipyramid for 3 h at 37 °C. Scale bar, 25 μm. (C) CLSM images of MCSs at the scanning depth from 10 to 90 μm after incubation with Au@mesoPt sphere, Au@mesoPt rod, and Au@mesoPt bipyramid for 1 h. Scale bar, 100 μm. (D) Blood clearance profiles in nude mice (*n* = 5) after intravenous injection of 100 μl of NPs (Au@mesoPt sphere, Au@mesoPt rod, and Au@mesoPt bipyramid) at an identical concentration of 50 nM kg^−1^ for 3 h. (E and F) Blood clearance (CL) rate (C), distribution half-lives (*t*_1/2α_), and elimination half-lives (*t*_1/2β_) (D) of Au@mesoPt sphere, Au@mesoPt rod, and Au@mesoPt bipyramid at 3 h following injection. Experiments were repeated 3 times, and the data are expressed as the mean ± SEM. *P < 0.05, **P < 0.01, ***P < 0.001.

Cytocompatibility is an essential prerequisite for the use of biomaterials. The cytotoxicity of the Au@mesoPt NPs was examined using a normal human embryonic kidney cell line (293T) and a human pancreatic cancer cell line (Aspc-1). Notably, after incubation with any Au@mesoPt NP, cell viabilities were still over 80%, regardless of the concentration used (0.5 to 10 pM; Fig. S4A and B). To assess the cellular uptake efficiency of the NPs, Cy5.5 surface-modified variants were developed, which exhibited intense fluorescence with increasing concentration (Fig. S4C and D). Consequently, flow cytometry results demonstrated that all Au@mesoPt NPs were taken up by Aspc-1 cells in a time-dependent manner (Fig. 3A and Fig. S4E), with the bipyramid morphology displaying the highest uptake after 6 h (39.31%, cf. rod: 26.05%, sphere: 13.06%). Immunofluorescence assays were used to assess the in vitro distribution of Au@mesoPt NPs, with time-lapse CLSM images showing that intracellular fluorescence intensity in cocultured Aspc-1 cells increased in a time-dependent manner. Again, fluorescence intensity for the Au@mesoPt bipyramid group was significantly and uniformly higher than that of Au@mesoPt rod or Au@mesoPt sphere groups (Fig. 3B and Fig. S4F to H), indicating enhanced cellular uptake. This reasonable reason might be due to that NPs with sharp edges and corners (i.e., bipyramid, but not spheroid) were capable of piercing cell and lysosome membranes, resulting in rapid accumulation and slower elimination, which had been reported in previous work [39]. Therefore, the higher cellular uptake of Au@mesoPt bipyramid NPs was most likely attributable to their unique structural characteristics.

To further investigate the therapeutic potential of the 3 NPs, MCSs derived from Aspc-1 cells were employed as in vitro 3D tumor models. Specifically, CLSM Z-stack scanning of cancer cell spheroids co-incubated with either sphere, rod, or bipyramid Au@mesoPt NPs enabled an assessment of tissue penetration through quantitative fluorescence intensity measurements. Consequently, the Au@mesoPt bipyramid NP treatment was observed to exhibit the greatest penetration depth (10 to 90 μm, with the outer edge of the MCSs defined as 0 μm), whereas the fluorescence signals for the Au@mesoPt rod and Au@mesoPt sphere groups were much weaker and mainly distributed at the periphery (Fig. 3C). Furthermore, the fluorescence intensity of bipyramid group was substantially higher than that of the rod and sphere groups under identical layer (Fig. S4I). It was suspected that the same intracellular mechanisms of absorption detailed above might be operative in this intercellular model, indicating that (in particular) the bipyramid geometry promoted transfer between adjacent cells or across the extracellular matrix.

### In vivo biological behaviors of Au@mesoPt NPs

To further ensure the biocompatibility of the Au@mesoPt NPs, a group of nude mice was subjected to intravenous dosing of Au@mesoPt NPs (50 nM kg^−1^ per mouse), with routine blood, serum, and histochemical tissue screening being subsequently performed after 14 d. Pointedly, blood biochemical indexes from the 3 Au@mesoPt NPs groups showed no significant abnormality when compared to the control group (Fig. S5A). Also, no obvious inflammation and necrosis were observable in any of the major organs [evaluated by hematoxylin and eosin (H&E) staining; Fig. S5B].

The pharmacokinetic profiles of any NP are essential factors that will determine whether they are suitable for biomedical or therapeutic applications [40]. As such, the concentration of gold and platinum (which provided a convenient indicator of Au@mesoPt levels) in plasma as a function of time was illustrated in Fig. 3D. The blood clearance profiles showed that, at 3 h following injection, Au@mesoPt bipyramid (30.90 ± 4.21%) possessed a significantly higher relative concentration than either the rod (6.41 ± 2.83%) or sphere (3.20 ± 1.62%) morphologies (Fig. 3D), indicating an increased blood circulation time. The corresponding clearance (CL) rate of Au@mesoPt bipyramid (30.71 ± 3.72 ml h^−1^ kg^−1^) was therefore also lower than that of Au@mesoPt rod (60.70 ± 11.43 ml h^−1^ kg^−1^) and Au@mesoPt sphere (90.13 ± 13.42 ml h^−1^ kg^−1^) (Fig. 3E). Also, the distribution half-lives (*t*_1/2α_) and elimination half-lives (*t*_1/2β_) of Au@mesoPt bipyramid (*t*_1/2α_: 37.21 ± 3.02 min; *t*_1/2β_: 4,057.81 ± 142.83 min) were higher than those of Au@mesoPt rod (*t*_1/2α_: 9.64 ± 1.82 min; *t*_1/2β_: 73.22 ± 1.21 min) and Au@mesoPt sphere (*t*_1/2α_: 13.2 ± 0.6 min; *t*_1/2β_: 45.6 ± 1.8 min) (Fig. 3F). The corresponding area under the curve (AUC_0–3h_) of Au@mesoPt bipyramid (0.50 ± 0.04 mg ml^−1^ h^−1^) was also larger than that of Au@mesoPt rod (0.28 ± 0.03 mg ml^−1^ h^−1^) and Au@mesoPt sphere (0.13 ± 0.01 mg ml^−1^ h^−1^) (Fig. S5C). The mean residence time (MRT_0–3h_) of Au@mesoPt bipyramid (1.10 ± 0.11 h) was longer than that of Au@mesoPt rod (0.91 ± 0.13 h) and Au@mesoPt sphere group (0.60 ± 0.10 h) (Fig. S5D). Taken together, these results indicated that Au@mesoPt bipyramid remained circulating in the blood for a longer period of time than either the spherical and rod-like congeners, most likely as a result of reduced clearance (most likely renal, but also possibly hepatic, etc.). Another possible reason could be that NPs with a high aspect ratio and anisotropy, such as bipyramid NPs, tend to accumulate along the margin of the blood flow, where the velocity is slow, leading to slower clearance. Conversely, non-anisotropic NPs, like rod-shaped and spherical NPs, as well as NPs with a low aspect ratio, such as spherical NPs, are distributed in the center of the blood flow, where the velocity is fast, resulting in faster clearance [30].

**Fig. 4.**
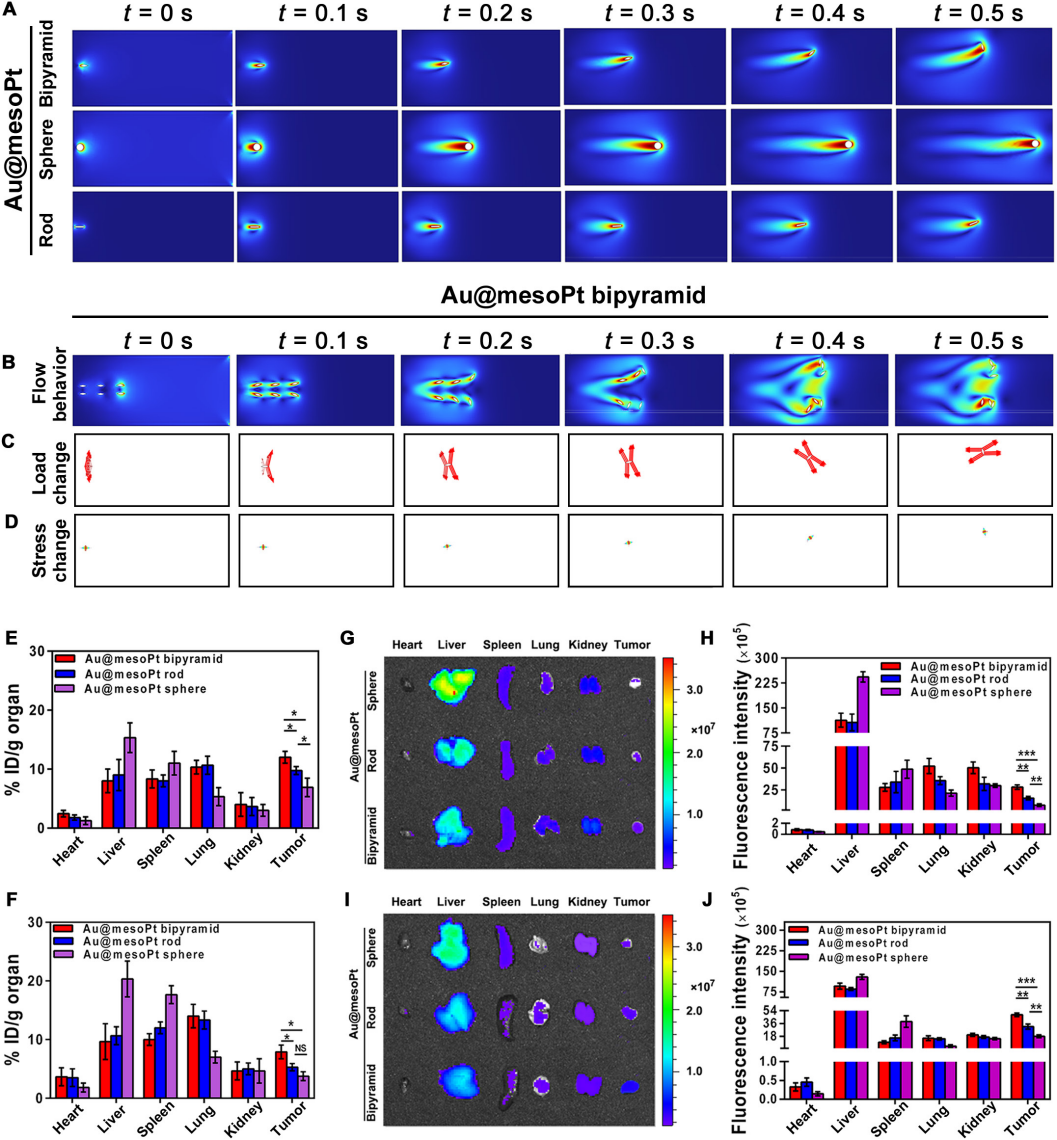
The biological performance of Au@mesoPt NPs. (A) Flow behavior of single NP for Au@mesoPt sphere, Au@mesoPt rod, or Au@mesoPt bipyramid under the same boundary conditions from 0 to 0.5 s simulated by COMSOL Multiphysics simulation software. (B) Numerical simulation of flow behavior for multiple Au@mesoPt bipyramid NPs in blood from 0 to 0.5 s. (C and D) Numerical simulation of load change (C) and stress change (D) of Au@mesoPt bipyramid in blood flow from 0 to 0.5 s. (E and F) Biodistribution of Au@mesoPt sphere, Au@mesoPt rod, and Au@mesoPt bipyramid in nude mice bearing Aspc-1 tumors at 12 h (E) and 24 h (F) post-injection of NPs (*n* = 5, 50 nM kg^−1^). (G to J) Ex vivo NIR fluorescence imaging and quantification of the fluorescence intensity of major organs (heart, liver, spleen, lung, kidney, and tumor) from nude mice bearing Aspc-1 tumors injected with Cy5.5-modified NPs (*n* = 5, 100 μl per mouse, 50 nM kg^−1^) for 12 h (G and H) and 24 h (I and J). Experiments were repeated 3 times, and the data are expressed as the mean ± SEM. *P < 0.05, **P < 0.01, ***P < 0.001.

To further verify the above conjecture, 2D numerical models were established for simulating the flow behaviors of 3 Au@mesoPt NPs in blood vessel (Fig. S1E and F). Primarily, based on the existing characteristics data, the key parameters of Au@mesoPt NPs had been calculated, including Young’s modulus *E*: 78.5 GPa, density *ρ*: 19.32 g/cm^3^, Poisson’s ratio ν:0.42, and the blood flow velocity *u*: 14 cm/s. The flow behavior of single Au@mesoPt NP under the same boundary condition from 0 to 0.5 s was then simulated (Fig. 4A). After starting to move, Au@mesoPt bipyramid flipped quickly toward the upper wall and then moved steadily with strong adhesion, while Au@mesoPt sphere moved forward with slight oscillations up and down rather than obvious adhesion to the upper wall. Moreover, the movement behavior of Au@mesoPt rod was similar but worse to bipyramid counterpart in flow rate and wall adhesion performance. Similar results were also observed in the simulated model of multiple Au@mesoPt bipyramid NPs (Fig. 4B). Two possible reasons could be attributed to this phenomenon: (a) Shape factors. The specific morphology (high aspect ratio and irregular surface) provided Au@mesoPt bipyramid a stable fulcrum to increase sliding friction when contacting with the blood vessel wall, helping move along edge easier. (b) Fluid dynamics factors. In flowing vessel, the flow velocity of blood generally decreased progressively from the center to the edge (also referred to as velocity profile or velocity gradient). The momentum transferred among fluid layers of blood flow might propel Au@mesoPt bipyramid to adhere to the edge due to the high blood viscosity.

The load that Au@mesoPt bipyramid suffered in 2D planar vessel model was further analyzed (Fig. 4C). Au@mesoPt bipyramid was subjected to a large load difference from the entrance (high load) to the export (low load), which rendered NP powerful driving force to move forward. During the process of movement, the gradually increased load difference between the upside (load decreased) and downside (load increased) of the Au@mesoPt bipyramid led to its wall approach, and following stable movement along the blood wall while NP load in all directions plateaued. The stress change of Au@mesoPt bipyramid during flowing was also explored (Fig. 4D). The upside stress of NP obviously concentrated from 0 to 0.2 s. Afterward, the downside stress of Au@mesoPt bipyramid began to strengthen in the process of near the upper wall. Finally, the overall stress distribution of NP became uniform when transforming into a steady motion state.

Given the longer circulation time of Au@mesoPt bipyramid, we further hypothesized that its accumulation in tumor tissues might be superior to that of the sphere or rod morphologies. To verify this premise, ICP-AES analysis and NIR fluorescence imaging were applied to assess the distribution of the 3 NPs in the Aspc-1 pancreatic cancer xenograft tumor model. After 12- or 24-h administration by injection, ICP-AES analysis showed that Au@mesoPt bipyramid accumulated more in tumor tissue than Au@mesoPt rod or Au@mesoPt sphere (Fig. 4E and F). This was further supported by the results of ex vivo NIR fluorescence imaging (Fig. 4G to J). Therefore, it could be concluded that Au@mesoPt bipyramid displayed stronger tumor permeability than Au@mesoPt sphere and Au@mesoPt rod, which was consistent with the in vitro findings. The reason for the superior tumor accumulation ability of Au@mesoPt bipyramid was, at present, unclear, but very likely follows the following [30–32]: (a) Shape-dependent penetration capability into the tumor microenvironment. The geometry of NPs, particularly the bipyramidal structure, can mitigate mechanical hindrances imposed by extracellular matrix components, thereby facilitating deeper penetration into tumor tissues. (b) Optimized utilization of the enhanced permeability and retention (EPR) effect. Au@mesoPt bipyramidal NPs, owing to their prolonged circulation time, maintain a higher concentration in the bloodstream, which extends their opportunity to interact with the interstitial spaces of tumor vasculature endothelial cells. Concurrently, the mesoporous platinum shell coating on the bipyramidal surface may reduce the likelihood of recognition and clearance by the reticuloendothelial system through modulation of surface charge or ligand functionalization. This dual mechanism indirectly supports both their circulatory stability and efficient exploitation of the EPR effect. (c) Synergistic passive targeting mediated by the tumor microenvironment. The bipyramidal geometry may enhance passive targeting efficacy by influencing interactions with tumor cells—for instance, via steric hindrance or receptor binding efficiency. Sharp tips on the bipyramidal structure could promote “anchoring” of NPs within tumor cell interstices, whereas spherical or rod-shaped NPs are more prone to plasma protein adsorption, leading to rapid clearance during circulation. This mechanism further corroborates the synergistic regulation of active/passive targeting by NP shape. It was fortuitous that this behavior was tumor specific, as the increased blood circulation time and lack of discernible specific organ accumulation would suggest that geometric properties do not promote uptake into, or transmission across, healthy tissues. This would suggest that other neoplasm-specific mechanisms were at play (perhaps pH-dependent absorption, or deposition in newly vascularized sites). Regardless, this area warrants further investigation.

### Anti-atherosclerosis therapy by Au@mesoPt NPs

**Fig. 5.**
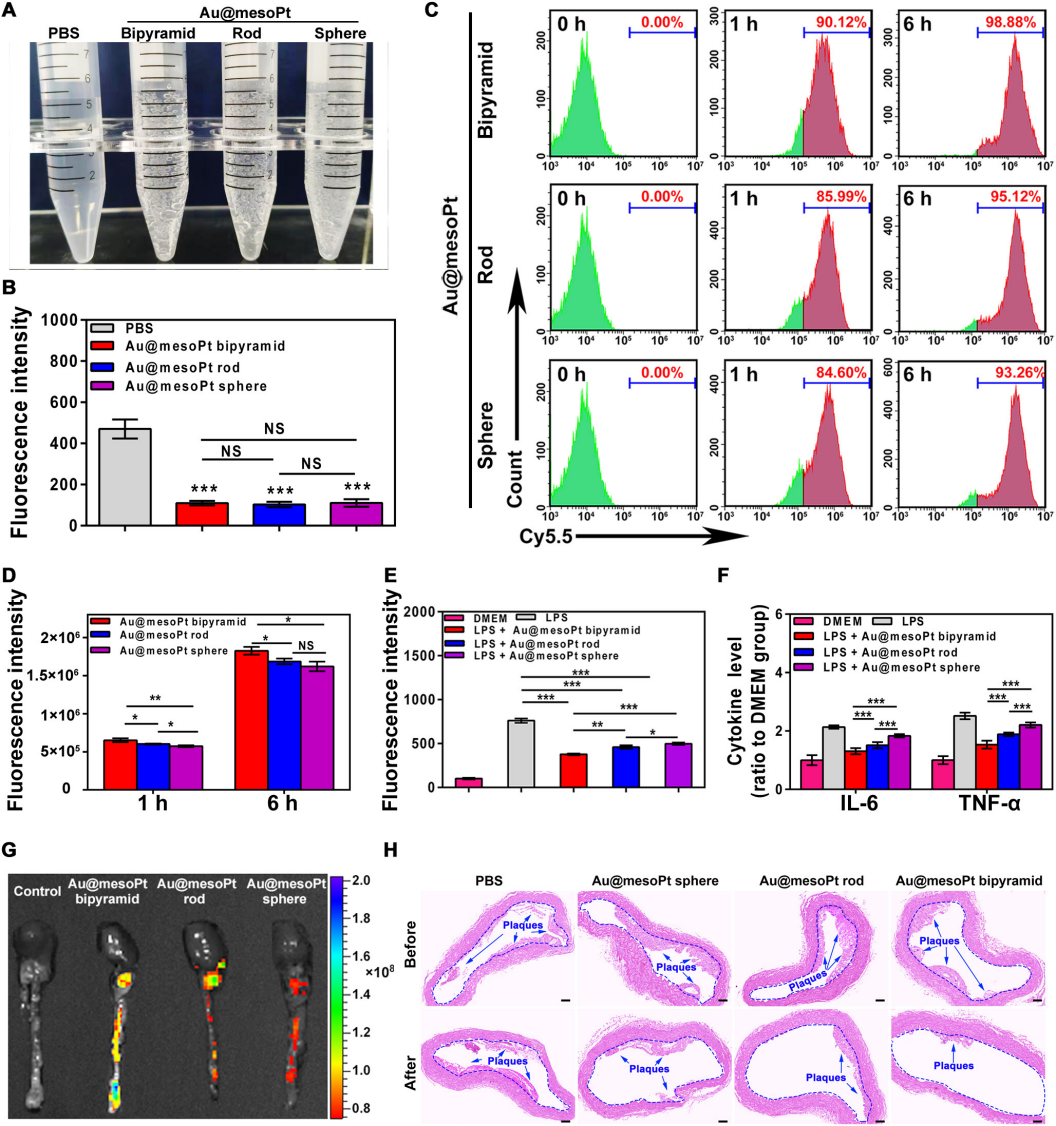
Atherosclerotic plaque accumulation and therapy effect of Au@mesoPt NPs. (A) Generation of oxygen gas bubbles in different groups. (B) Average fluorescence intensity of [Ru(dpp)_3_]Cl_2_-added mixture containing H_2_O_2_ and PBS, Au@mesoPt bipyramid, Au@mesoPt rod, or Au@mesoPt sphere. (C and D) Flow cytometry analysis of the uptake of Au@mesoPt sphere, Au@mesoPt rod, and Au@mesoPt bipyramid by Raw 264.7 cells at the indicated time (1 and 6 h). (E) DCFH-DA fluorescence intensity of DMEM- or LPS-treated Raw 264.7 cells in the presence of Au@mesoPt bipyramid, Au@mesoPt rod, and Au@mesoPt sphere. (F) Pro-inflammatory cytokine level (IL-6, TNF-α) in the supernatant of LPS-treated Raw 264.7 cells in the presence of DMEM, Au@mesoPt bipyramid, Au@mesoPt rod, and Au@mesoPt sphere. (G) Ex vivo NIR fluorescence imaging of the aorta treated with PBS, Au@mesoPt bipyramid, Au@mesoPt rod, and Au@mesoPt sphere. (H) H&E staining of aortas from ApoE^–/–^ mice treated with PBS, Au@mesoPt sphere, Au@mesoPt rod, and Au@mesoPt bipyramid before and after 3 weeks of administration. Atherosclerotic plaques were indicated by the dark blue arrow in the blue dashed circle. Scale bar, 200 μm. Experiments were repeated 3 times, and the data are expressed as the mean ± SEM. *P < 0.05, **P < 0.01, ***P < 0.001.

With considerable evidence suggesting that Au@mesoPt NPs (and especially the bipyramidal morphology) specifically target tumor cells and tissues, we envisioned that these materials would be applicable to treatment researches in different disease models. The overproduction of ROS, specifically as a result of elevated macrophage activity, plays a critical role in deteriorating atherosclerotic plaques [41]. Notably, Pt-based NPs had proven to be an effective ROS scavenger both in vitro and in vivo [42], suggesting that they might be applicable in this setting*.* The “catalase-like” activity of the Au@mesoPt bipyramid, rod, and sphere morphologies was therefore evaluated with a robust (albeit simple) chemical assay. After adding Au@mesoPt NPs to an aqueous H_2_O_2_ solution, obvious oxygen bubbles (Fig. 5A) and elevated O_2_ levels (indicated by the fluorescence intensity of an [Ru(dpp)_3_]Cl_2_ probe; Fig. 5B) were observed, indicating the catalysis of peroxide disproportionation. Notably, there was no significant difference in O_2_ levels between the 3 NPs groups, suggesting no morphology-dependent behavior.

Following the promising demonstration of antioxidant properties, the biocompatibility and cellular uptake of Au@mesoPt NPs in Raw 264.7 macrophage was evaluated. Bipyramid, rod, and sphere topologies demonstrated low cytotoxicity (Fig. S5E) and were all efficiently taken up by Raw 264.7 cells, as confirmed by flow cytometry (Fig. 5C and D). More importantly, the percentage of cells with internalized Au@mesoPt bipyramid NPs was significantly higher than that of the rod and sphere counterparts. It is speculated that such discrepancies in cellular uptake may endow bipyramid Au@mesoPt NPs with superior ROS clearing ability.

Inspired by the excellent biocompatibility, catalase-like activity, and suitable cellular uptake properties, the ability of Au@mesoPt NPs to eliminate ROS in both Raw 264.7 macrophage and an atherosclerosis animal model was next assessed*.* LPS is well known to induce elevated ROS levels via promotion of a pro-inflammatory response in Raw 264.7 cells [43]. As such, the fluorescence intensity of DCFH-DA (an indicator of intracellular ROS levels) in LPS-treated Raw 264.7 cells was significantly elevated above the control. It is pleased to observed, however, that the presence of Au@mesoPt NPs significantly inhibited this response. Moreover, Au@mesoPt bipyramid possessed the strongest inhibition ability, indicating the strongest ROS eliminating capacity, which might be attributed to the higher cellular uptake efficiency of Au@mesoPt bipyramid (Fig. 5E). Notably, ROS can also induce the production of pro-inflammatory cytokines, which are critical to the progression of atherosclerotic plaques [44]. Remarkably, the levels of pro-inflammatory cytokines (specifically IL-6 and TNF-α) in the supernatant of LPS-treated Raw 264.7 cells were obviously reduced in the presence of Au@mesoPt NPs, with Au@mesoPt bipyramid displaying the greatest pro-inflammatory cytokine inhibition effect (Fig. 5F).

Considering the outstanding ROS eliminating and pro-inflammatory cytokine release inhibition capacity of Au@mesoPt NPs, the therapeutic effect of these materials in an atherosclerosis animal model was evaluated. The accumulation of the bipyramid, rod, and sphere morphologies in aortic atherosclerotic plaques was first assessed by ex vivo NIR fluorescence imaging. Punctuated and streaked signals were observed in the aortas, with fluorescence intensities again being greatest in the bipyramid group, followed by rod and sphere groups (Fig. 5G). To further verify the fluorescence signals, ex vivo aortas were collected and the presence of plaques was confirmed by histochemical staining. Following this, the therapeutic effect of Au@mesoPt bipyramid, Au@mesoPt rod, and Au@mesoPt sphere in developed atherosclerosis plaque ApoE^–/–^ mice was assessed. Compared to the PBS group, H&E staining results revealed that the size of plaques in the aorta shrunk significantly in the Au@mesoPt bipyramid treated group, over and above that of the Au@mesoPt rod and Au@mesoPt sphere groups (Fig. 5H). These results could be explained by the longer blood circulation time of Au@mesoPt bipyramid, coupled with the improved ROS scavenger ability, leading to the alleviation of ROS-based inflammation reactions. The above results are indeed consistent with the results of studies reporting that scavenging ROS to alleviate disease progression involves excessive oxidation [45].

### Synergic RT by Au@mesoPt NPs

**Fig. 6.**
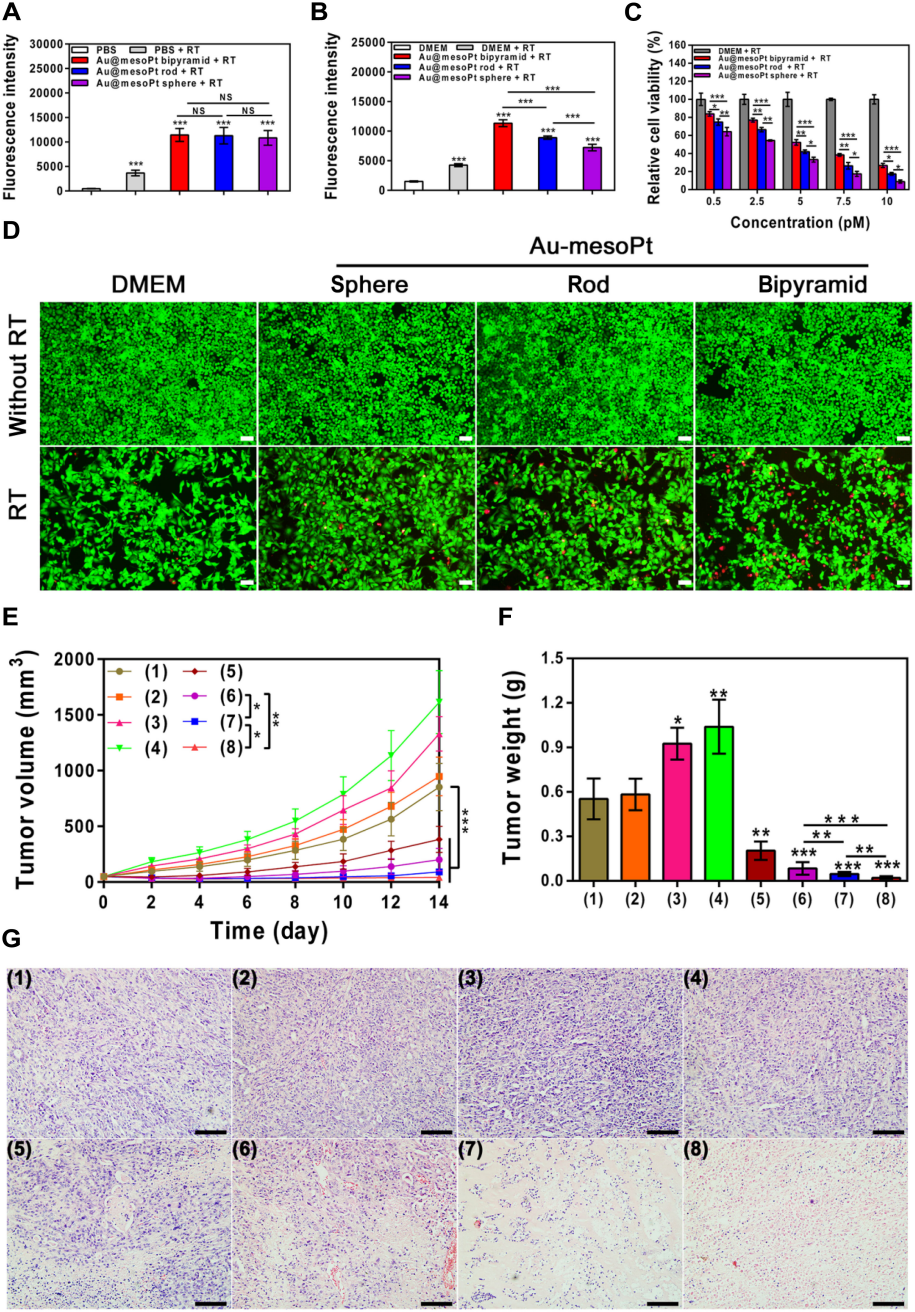
RT sensitization effect of the Au@mesoPt NPs. (A) Generation of singlet oxygen (^1^O_2_) in different agents PBS, Au@mesoPt sphere (10 pM), Au@mesoPt rod (10 pM), and Au@mesoPt bipyramid (10 pM)] with or without x-ray irradiation at 8 Gy measured by flow cytometry. (B) Generation of ROS in Aspc-1 cells treated by different agents [DMEM, Au@mesoPt sphere (10 pM), Au@mesoPt rod (10 pM), and Au@mesoPt bipyramid (10 pM)] with or without x-ray irradiation at 8 Gy measured by flow cytometry. (C and D) Relative cell viability (C) of Aspc-1 cells treated with DMEM or Au@mesoPt NPs (sphere, rod, and bipyramid, 0.5 to 10 pM) and cell live/dead staining (D) of Aspc-1 cells incubated with DMEM and Au@mesoPt NPs (sphere, rod, and bipyramid, 10 pM) and then with or without x-ray irradiation (8 Gy). Scale bar, 50 μm. (E and F) Tumor volumes (E) and tumor weights (F) at the end point of the following treatments (*n* = 5): (1) PBS group, (2) Au@mesoPt sphere group, (3) Au@mesoPt rod group, (4) Au@mesoPt bipyramid group, (5) RT group, (6) Au@mesoPt sphere + RT, (7) Au@mesoPt rod + RT, and (8) Au@mesoPt bipyramid + RT group. The tumor sites of the appropriate groups (5, 6, 7, and 8) were treated with x-ray irradiation (8 Gy) every other day for 3 times. (G) H&E-stained images of tumor tissues with different treatments at 14 d. Scale bar, 200 μm. Experiments were repeated 3 times, and the data are expressed as the mean ± SEM. *P < 0.05, **P < 0.01, ***P < 0.001.

The depletion of ROS can indeed effectively retard the progression of disorders characterized by heightened oxidative stress, such as atherosclerosis, but for some diseases with excessive oxidative stress, such as solid tumors, including pancreatic cancer, the augmentation of ROS levels can also restrain their occurrence and development [46]. Compared with other stimulus-responsive therapeutic modalities (e.g., photothermal or magnetothermal therapies), x-ray-based RT offers several distinct advantages [47–49]: (a) Its strong penetrating capability enables noninvasive, deep-tissue tumor treatment with precise local control; (b) it demonstrates superior performance in complex anatomical sites (e.g., pancreatic cancer) where light- or magnetic field-based approaches face technical challenges; (c) it can induce immunogenic cell death to activate the host immune system and enhance therapeutic efficacy; and (d) when combined with intensity-modulated RT and image-guided techniques, it apparently minimizes damage to surrounding healthy tissues. It has been reported that hypoxia relief and increased generation of more cytotoxic ROS (^1^O_2_ etc.) in tumors can visibly enhance the effectiveness of RT [50], an established method for treating solid tumors such as pancreatic cancer and breast cancer. Furthermore, inspired by nanomaterials containing high-atomic-number (*Z*) elements (e.g., Au- or Pt-based NPs) as radiosensitizers to deposit radiation ray (e.g., x-ray) energy within tumors and promote treatment efficacy [20], and Au@mesoPt NPs’ superior biological profile and catalase-like ability of convert H_2_O_2_ to O_2_, the as-prepared Au@mesoPt NPs were elected to investigate their potential radiosensitization utilization in RT of pancreatic cancer. Firstly, the in vitro radiosensitization effect of the 3 NPs was evaluated. Solutions containing 10 pM Au@mesoPt NPs experienced substantial SOSG fluorescence intensity increases but no obvious difference among one another after 8-Gy x-ray irradiation (bipyramid: 11,433 ± 1,320, rod: 11,290 ± 1,681, sphere: 10,833 ± 1,501), much more so than PBS group (470 ± 46) or PBS + RT group (3,666 ± 568), which indicated the 3 Pt-based NPs indeed could absorb x-ray to strengthen the production of ^1^O_2_ (Fig. 6A). Concurrently, compared to the DMEM group (1,506 ± 90) or DMEM + RT (4,266 ± 251), DCFH-DA signal in Aspc-1 cells incubated with Au@mesoPt NPs also progressively increased (sphere: 7,233 ± 550, rod: 8,900 ± 268, bipyramid: 11,346 ± 578) after 8-Gy x-ray irradiation, suggesting the higher level of ROS production (Fig. 6B). The difference of intracellular ROS generation may be ascribed to the distinct cellular uptake efficiency of the 3 NPs. Following this, CCK-8 and calcein-AM/propidium iodide (PI) assay were deployed to quantify the RT efficacy in Aspc-1 cells. CCK-8 assays demonstrated that relative cell viabilities in NP-treated groups displayed a dose-dependent decrease (over a 0.5 to 10 pM range) after 8-Gy x-ray irradiation (Fig. 6C), with the optimal RT efficacy ranking (Au@mesoPt bipyramid + RT group > Au@mesoPt rod + RT group > Au@mesoPt sphere + RT group). As demonstrated in Fig. 6D by live/dead staining (calcein-AM/PI), a progressive increase in cancer cell death was observed across the following groups compared to the nonirradiated groups: DMEM + RT, and further enhanced in groups treated with Au@mesoPt NPs of distinct morphologies (bipyramidal, rod-like, and spherical) + RT. These collective findings reveal a morphology-dependent radiosensitization effect conferred by the Au@mesoPt NPs. The underlying mechanisms involve the following [20,50]: (a) Alleviation of tumor hypoxia. The platinum (Pt) component within Au@mesoPt NPs catalyzes the decomposition of intracellular ROS to generate oxygen, thereby ameliorating hypoxic conditions and enhancing radiosensitivity. Notably, among the 3 morphologies, bipyramidal Au@mesoPt NPs exhibited the highest cellular internalization, resulting in the most significant hypoxia alleviation and thus optimal radiosensitization efficacy. (b) Augmented photoelectric effect for enhanced radiation energy deposition. When ionizing radiation from RT interacts with the high-atomic-number (*Z*) elements gold (Au) and platinum (Pt) within Au@mesoPt NPs, a pronounced photoelectric effect occurs. During this process, metal atoms absorb photon energy, ejecting core–shell electrons (Auger or photoelectrons) that generate high-energy secondary electrons. These secondary electrons induce water radiolysis, producing abundant cytotoxic ROS, including hydroxyl radicals (•OH) and superoxide anions (O_2_^•−^) to inflict substantial oxidative cascade damage on cellular biomacromolecules, leading to DNA strand breaks, protein denaturation, and lipid peroxidation.

Evidence for a profound RT effect with our Au@mesoPt NPs compelled us to investigate its applicability in an in vivo nude mice model (Aspc-1 xenograft tumor; Fig. 6E and F). When compared to a PBS control, groups treated with Au@mesoPt NPs alone possessed significantly greater tumor volumes and weights, perhaps as a result of the Pt-based NPs alleviating hypoxia in the tumor microenvironment (H_2_O_2_ to O_2_) and therefore promoting growth. Notably, tumor volumes and weights were greater in the bipyramid group than in the rod and sphere groups, presumably as a result of greater accumulation and deeper penetration. Gratifyingly, tumor size was remarkably diminished and growth was significantly inhibited in groups subjected to the combined Au@mesoPt NPs + RT treatment. The superior performance of the bipyramid topology was again noted, displaying higher tumor-inhibiting and regrowth-inhibiting ability than either rod or sphere morphologies. From the H&E staining, larger necrotic zones were observable in tumor tissues, with size ranking in the order bipyramid > rod > sphere. No obvious necrosis was detected in the other groups (Fig. 6G). Finally, negligible weight fluctuations in all mice during treatment demonstrated its safety in vivo (Fig. S5F). These results indicated that Au@mesoPt NPs could significantly improve radiosensitivity, and Au@mesoPt bipyramid NPs were significantly higher than those of other treatment groups, which was attributed to the fact that Au@mesoPt bipyramid NPs were taken up more by cells.

## Conclusion

In this study, 3 morphologically distinct mesoporous Au–Pt nanoenzymes (Au@mesoPt NPs) were constructed using a facile ALD methodology by templating Pt deposition off a preformed Au core. These MMNs were then demonstrated to possess distinct morphology-dependent behavior in the treatment of atherosclerosis and RT for pancreatic cancer. Our study established Au@mesoPt bipyramid as the ideal MMNs for this purpose given its superior physical and biological properties, including enhanced cellular uptake, prolonged blood circulation time, and greater tumor penetration in an MCS. As a result, Au@mesoPt bipyramid NPs (more so than rod or sphere) showed remarkable RT-induced pancreatic tumor inhibition via radiosensitization effect and atherosclerotic plaque reduction by ROS neutralization. Although this study has successfully delineated the influence of Au@mesoPt NP morphology and catalytic properties on their biological behavior and therapeutic efficacy in atherosclerosis and pancreatic cancer models, there are still some challenges that need to be addressed during the process of clinical translation. The potential contributions of other physicochemical parameters intrinsic to Au@mesoPt NPs to the observed effects, along with the long-term systematic biological safety and underlying molecular mechanisms governing their specific roles, remain to be comprehensively elucidated in future investigations. Hence, further in-depth exploration is still necessary to reveal the long-term in vivo safety, more physiochemical parameter functions, as well as their potential molecular biological mechanisms of Au@mesoPt NPs in the above disease models. Overall, our investigation indicated that Au@mesoPt bipyramid NPs had the potential to serve as a “nanoplatform” in the treatment of diseases associated with oxidative stress. Previous researches consistently highlight the significant clinical translation potential of Au- or Pt-based nanomaterials, attributed to their favorable biocompatibility, enhanced catalytic activity, computed tomography imaging enhancement capabilities, photothermal effects, and photodynamic amplification properties [13–17]. Consequently, Au@mesoPt NPs demonstrate immense promise as novel drug delivery systems capable of enabling multimodal theranostic strategies within pathological contexts characterized by excessive oxidative stress, such as solid tumors, Alzheimer’s disease, and diabetes. Simultaneously, this work contributed to further the exploration of morphology-dependent effects in other metal-based nanomaterials.

## Ethical Approval

All animal experiments complied with the ARRIVE 2.0 guidelines and were carried out in accordance with the National Institutes of Health Guide for the Care and Use of Laboratory Animals [33,34]. All procedures involving animals were performed under the guidelines approved by the Committee on the Use of Live Animals for Teaching and Research of Jiangsu University (no. UJS-IACUC-AP-2024031411).

## Data Availability

All data can be provided as needed.
