# Supplementary material for: Facile Synthesis of Designer Shape-Defined Mesoporous Metal Nanoenzymes as Therapeutics for Diseases Involving Excessive Oxidative Stress
Source: Biomater Res. 2025 Sep 5;29:0251. doi: 10.34133/bmr.0251 (PMC12411696; doi:10.34133/bmr.0251)
Supplement: Supplementary 1 — Figs. S1 to S5 [file bmr.0251.f1.docx]

# Supplemental Materials

**Facile synthesis of designer shape-defined mesoporous** **metal** **nanoenzymes as** **therapeutics** **for** **diseases involving excessive oxidative stress**

Xiongfeng Cao^1,2,†^, Kun Chen^3,†,*^, Minjun Ji^4,†^, Xiang Liao^1,2,*^, Yanfang Liu^5,*^


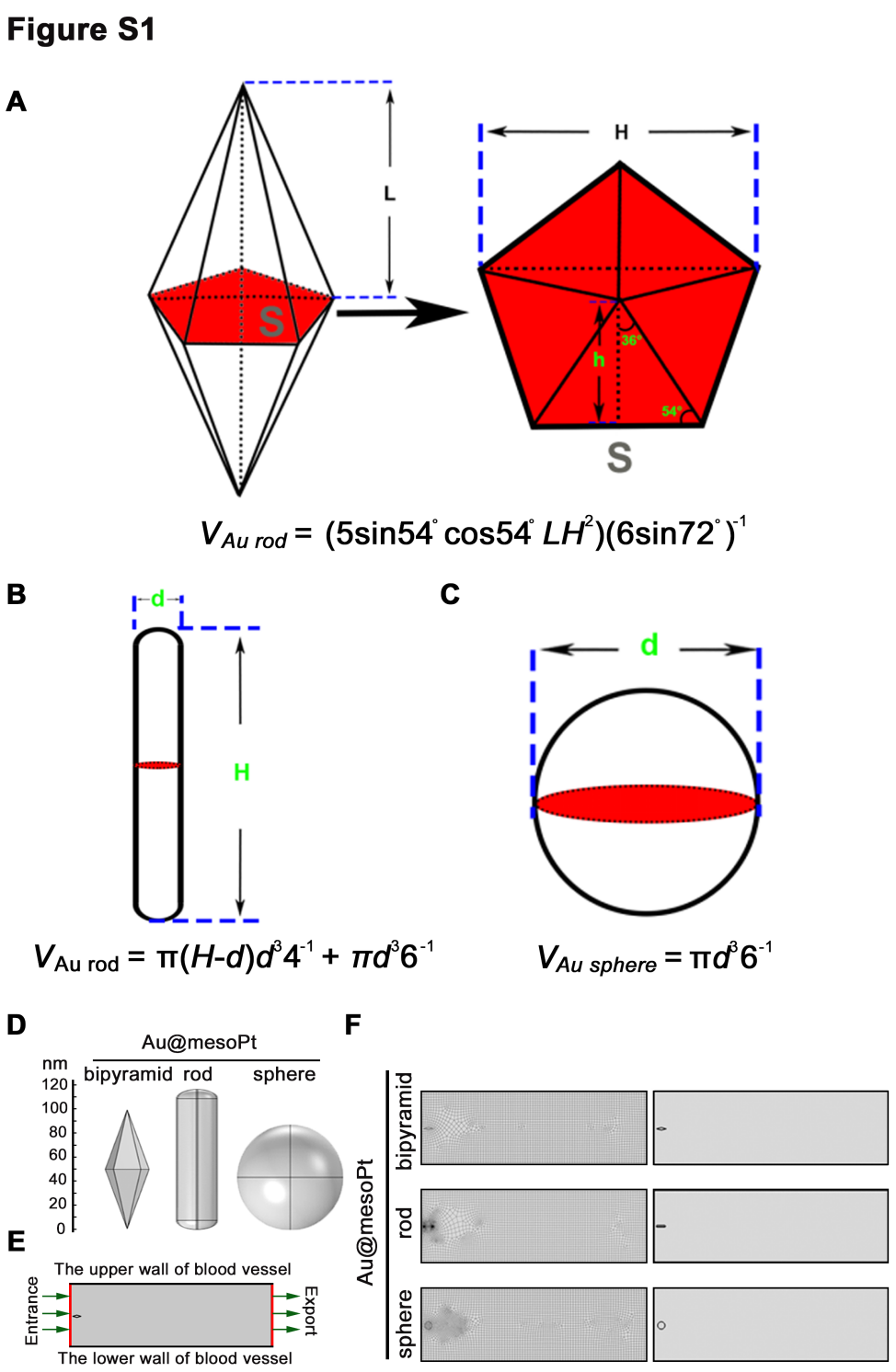


**Fig. S1.** Mathematical geometric models of NPs. (A-C) Mathematical geometric models and equations of Au bipyramid (A), Au rod (B), and Au sphere (C). (D) The geometric model of Au@mesoPt sphere, Au@mesoPt rod, or Au@mesoPt bipyramid. (E) The boundary and the initial conditions of Au@mesoPt NPs moving along blood vessel. (F) The mesh partitioning diagram of Au@mesoPt sphere, Au@mesoPt rod, or Au@mesoPt bipyramid.


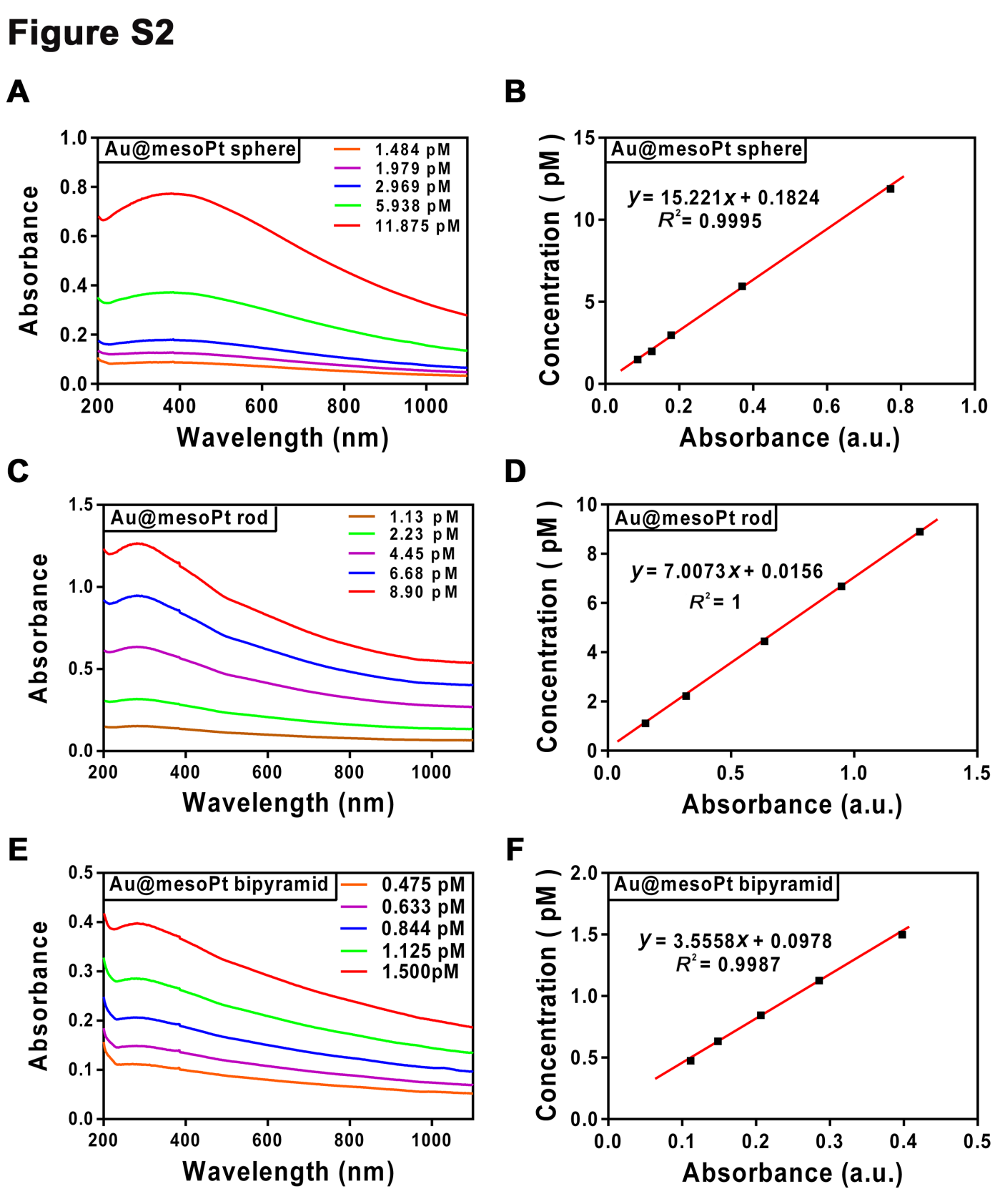


**Fig. S2.** Characterization of Au@mesoPt NPs. (A-F) The standard absorbance curves and UV-vis-NIR absorbance spectrum of Au@mesoPt sphere (A, B), Au@mesoPt rod (C, D), and Au@mesoPt bipyramid (E, F) at different concentrations.


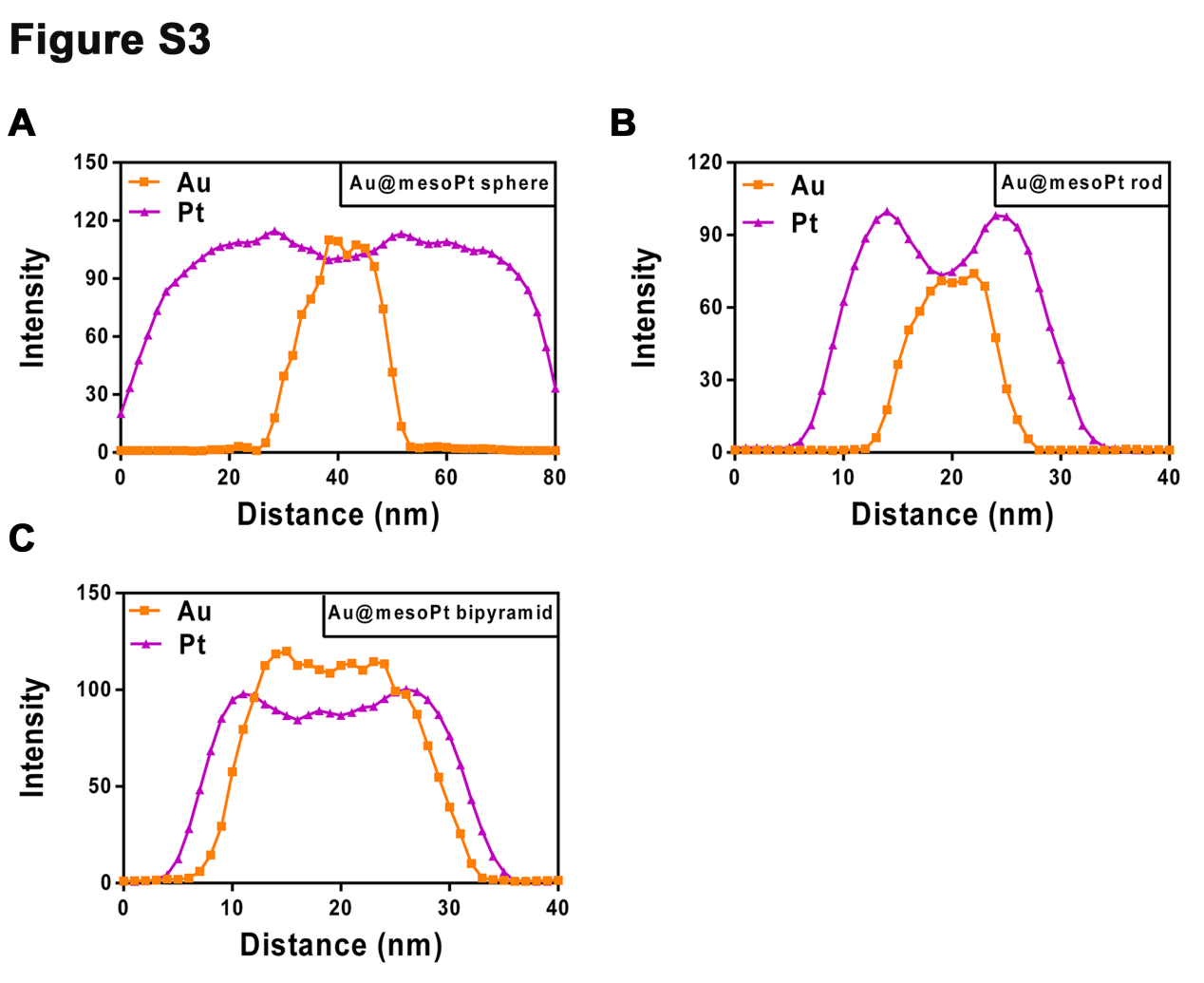


**Fig. S3.** The energy Dispersive X-ray (EDX) line profiles of Au@mesoPt sphere (A), Au@mesoPt rod (B) and Au@mesoPt bipyramid (C).


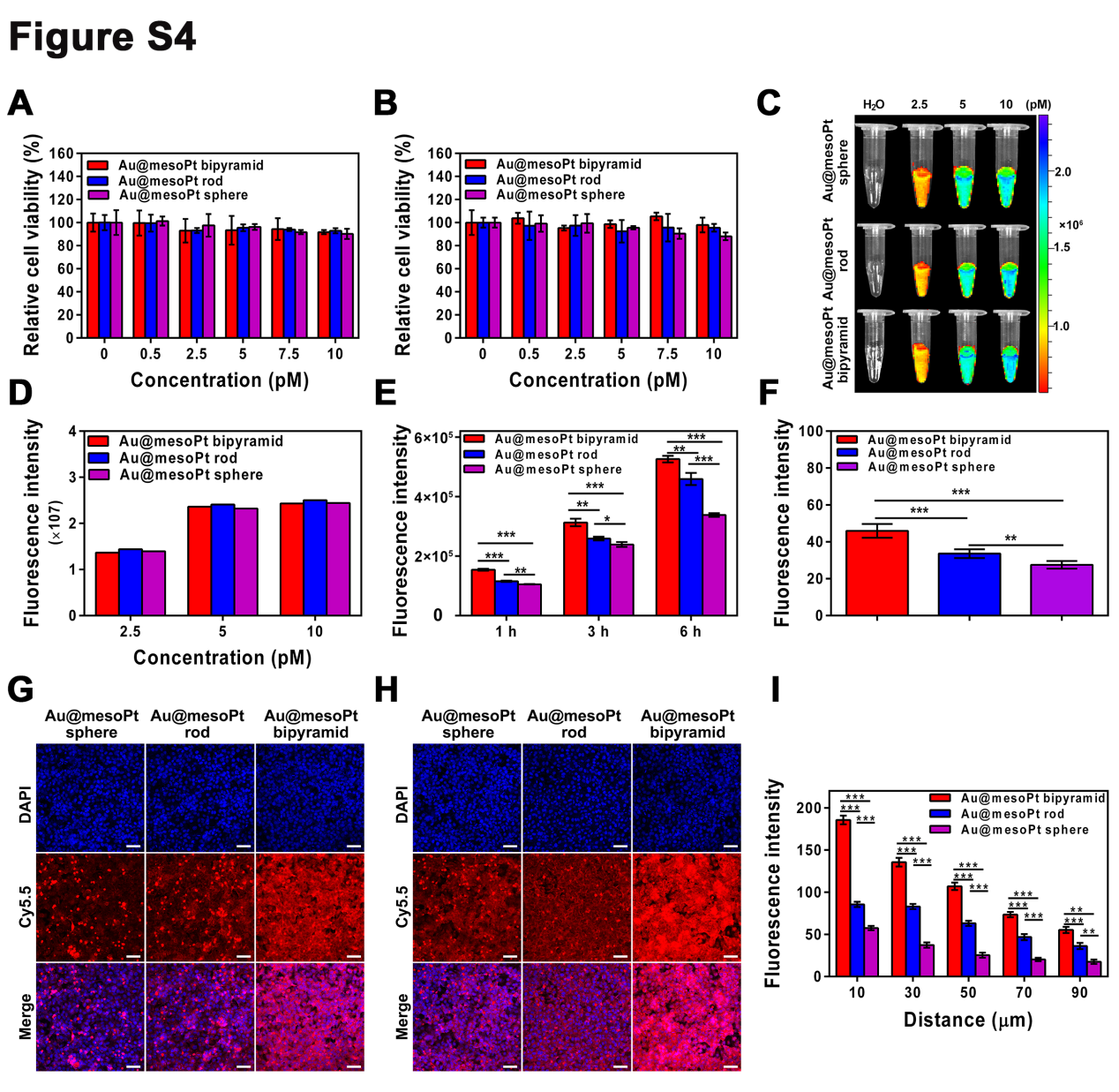


**Fig S4.** Biological behaviors of Au@mesoPt NPs. (A, B) Relative cell viability of Aspc-1 (A) and 293T (B) cells after incubation with Au@mesoPt sphere, Au@mesoPt rod and Au@mesoPt bipyramid for 48 h. (C, D) NIR fluorescence imaging (C) and mean fluorescence intensities (D) of the three Cy5.5-modified NPs at 2.5, 5 and 10 pmol L^-1^. (E) Mean fluorescence intensities of Aspc-1 cells after incubation with Au@mesoPt sphere, Au@mesoPt rod and Au@mesoPt bipyramid for the indicated time (1, 3, 6 h). (F) Fluorescence intensities of Aspc-1 cells after incubation with Au@mesoPt sphere, Au@mesoPt rod and Au@mesoPt bipyramid at 1 h. (G, H) Confocal laser scanning microscopy (CLSM) images of Aspc-1 cells after incubation with Au@mesoPt sphere, Au@mesoPt rod and Au@mesoPt bipyramid at 3 h (G) and 6 h (H). Scale bar is 25 µm. (I) Quantification of the fluorescence intensities of multicellular spheroids (MCSs) at the scanning depth from 10 to 90 µm after incubation with Au@mesoPt sphere, Au@mesoPt rod and Au@mesoPt bipyramid for 3 h. Experiments were repeated three times, and the data are expressed as the mean ± SEM. **P*<0.05, ***P*<0.01, ****P*<0.001.


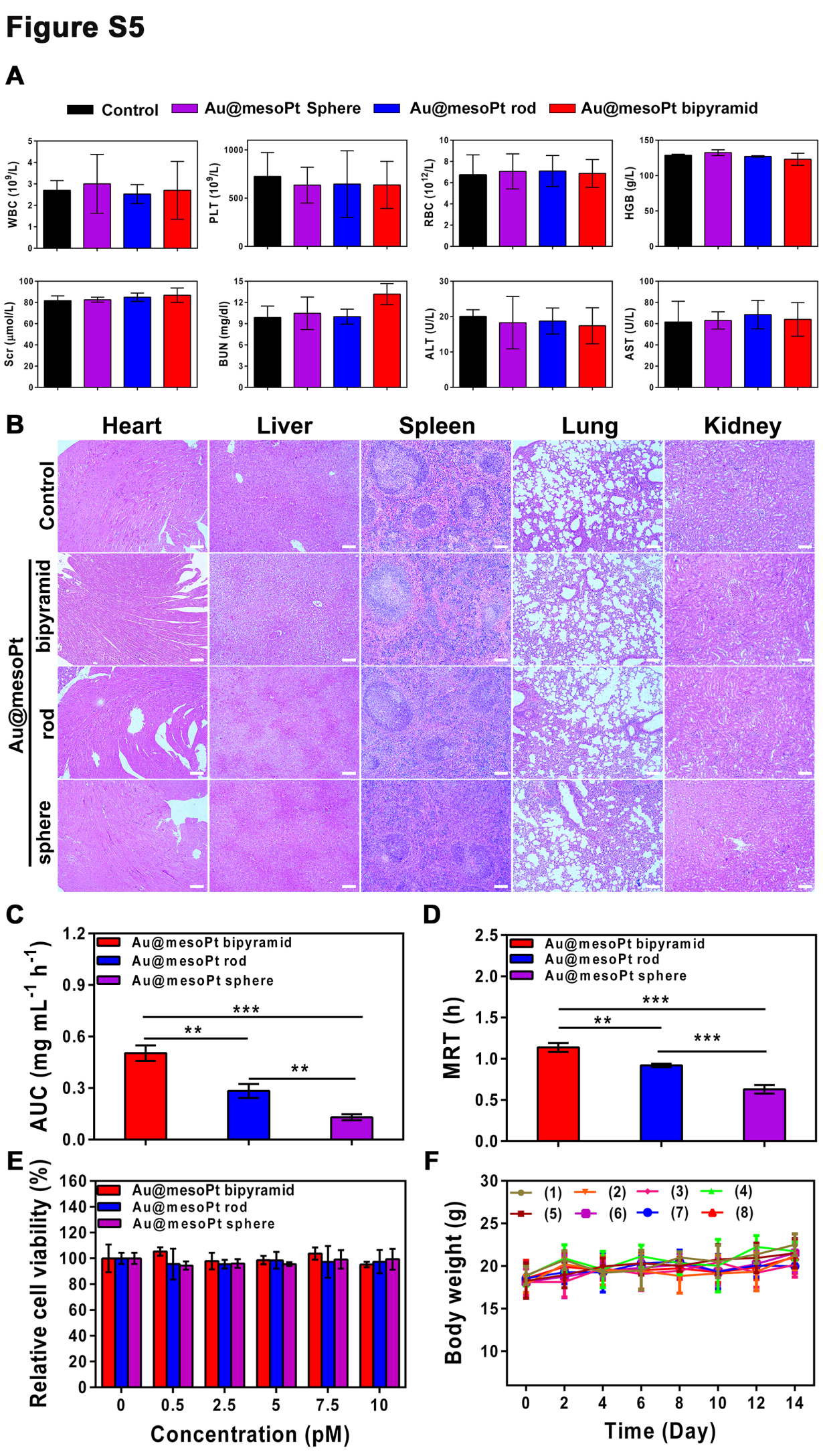


**Fig. S5.** *In vivo* biocompatibility and biological behaviors of Au@mesoPt NPs. (A) The blood biochemical indexes from nude mice treated with PBS, Au@mesoPt sphere, Au@mesoPt rod and Au@mesoPt bipyramid 14 days after administration. The blood biochemical indexes include white blood cell (WBC), platelet (PLT), red blood cell (RBC), hemoglobin (HGB), serum creatinine (Scr), blood urea nitrogen (BUN), alanine transaminase (ALT) and aspartate transaminase (AST). (B) Hematoxylin & Eosin (H&E) staining of major organs (heart, liver, spleen, lung and kidney) from nude mice treated with Au@mesoPt sphere, Au@mesoPt rod and Au@mesoPt bipyramid 14 days after administration. Scale bar is 20 μm. (C, D) Area under the curve (AUC_0-3h_) (C) and mean residence time (MRT_0-3h_) (D) of Au@mesoPt sphere, Au@mesoPt rod and Au@mesoPt bipyramid at 3 h following injection. (E) Relative cell viability of Raw264.7 cells after incubation with Au@mesoPt sphere, Au@mesoPt rod and Au@mesoPt bipyramid for 48 h. (F) Body weights at the end point following different treatments (n=5): (1) PBS group, (2) Au@mesoPt sphere group, (3) Au@mesoPt rod group, (4) Au@mesoPt bipyramid group, (5) RT group, (6) Au@mesoPt sphere + RT, (7) Au@mesoPt rod + RT, and (8) Au@mesoPt bipyramid + RT group, the tumor sites of the appropriate groups (5, 6, 7 and 8) were treated with X-ray irradiation (8 Gy) every other day for three times. Experiments were repeated three times, and the data are expressed as the mean ± SEM. **P*<0.05, ***P*<0.01, ****P*<0.001.
